# Active streets for children: The case of the Bogotá Ciclovía

**DOI:** 10.1371/journal.pone.0207791

**Published:** 2019-05-15

**Authors:** Camilo A. Triana, Olga L. Sarmiento, Alejandra Bravo-Balado, Silvia A. González, Manuel A. Bolívar, Pablo Lemoine, Jose D. Meisel, Carlos Grijalba, Peter T. Katzmarzyk

**Affiliations:** 1 Department of Public Health, School of Medicine, Universidad de los Andes, Bogotá, Colombia; 2 Healthy Active Living and Obesity Research Group, Children’s Hospital of Eastern Ontario Research Institute, Ottawa, Ontario, Canada; 3 Department of Industrial Engineering, Faculty of Engineering, Universidad de los Andes, Bogotá, Colombia; 4 Facultad de Ingeniería, Universidad de Ibagué, Ibagué, Colombia; 5 Pennington Biomedical Research Center, Baton Rouge, Louisiana, United States of America; Universite Cote d’Azur, FRANCE

## Abstract

**Introduction:**

The Ciclovía is a worldwide program in which streets are temporarily closed to motorized transport to create a space for recreation and outdoor play among children and adults. The aim of this study was to assess the associations between physical activity (PA), sedentary time (SED), body mass index and Ciclovía participation among children aged 9 to 13 years.

**Methods:**

All students in the 4^th^ and 5^th^ grades from the selected schools were invited to participate in the study. The study included 923 children. PA and SED were measured using waist-worn accelerometers, and height and weight were measured using standardized procedures. Ciclovía participation was self-reported. The analyses included multilevel linear, generalized mixed and generalized additive models.

**Results:**

The mean age of the sample was 10.1±0.7 years, and 49.5% were boys. In the last year, 46% of the children participated in the Ciclovía, and 34% reported participating frequently (at least once per month). No differences were found in the mean minutes of moderate-to-vigorous PA on weekdays between frequent Ciclovía users and sporadic and non-Ciclovía users (72 vs 69; p = 0.09). In contrast, frequent Ciclovía users had higher moderate-to-vigorous PA on Sundays than sporadic and non-Ciclovía users (65.6 vs 59.2; p = 0.01), specifically between the hours of 12:00 and 16:00. In addition, frequent Ciclovía users did not differ from the sporadic and non-Ciclovía users in SED (515.3 vs 521.3; p = 0.19). Frequent Ciclovía users had lower SED on Sundays than the sporadic and non-Ciclovía users (437.7 vs 456.5; p = 0.005). Additionally, frequent Ciclovía users were more likely to be overweight (28.3% vs 20.4% p = 0.01). We did not find differences in participation by sex, and low-to-middle income children were more likely to participate.

**Conclusions:**

The Ciclovías offer an innovative, inclusive recreational space and consequently provide opportunities to increase moderate-to-vigorous PA and reduce SED among children.

## Introduction

Childhood is undoubtedly a crucial period for acquiring healthy lifestyle habits[1]. Unfortunately, the majority of children worldwide are insufficiently active[2,3] and spend excessive time in sedentary behaviors[3,4]. Physical inactivity and sedentary behavior have been associated with obesity[5], poor cardio-metabolic health[6], and poor psychosocial health[7]. Currently, there is global concern regarding the need to build societies in which children can play and choose to participate in physical activity (PA) and reduce their sedentary time (SED)[8,9].

**Fig 1:**
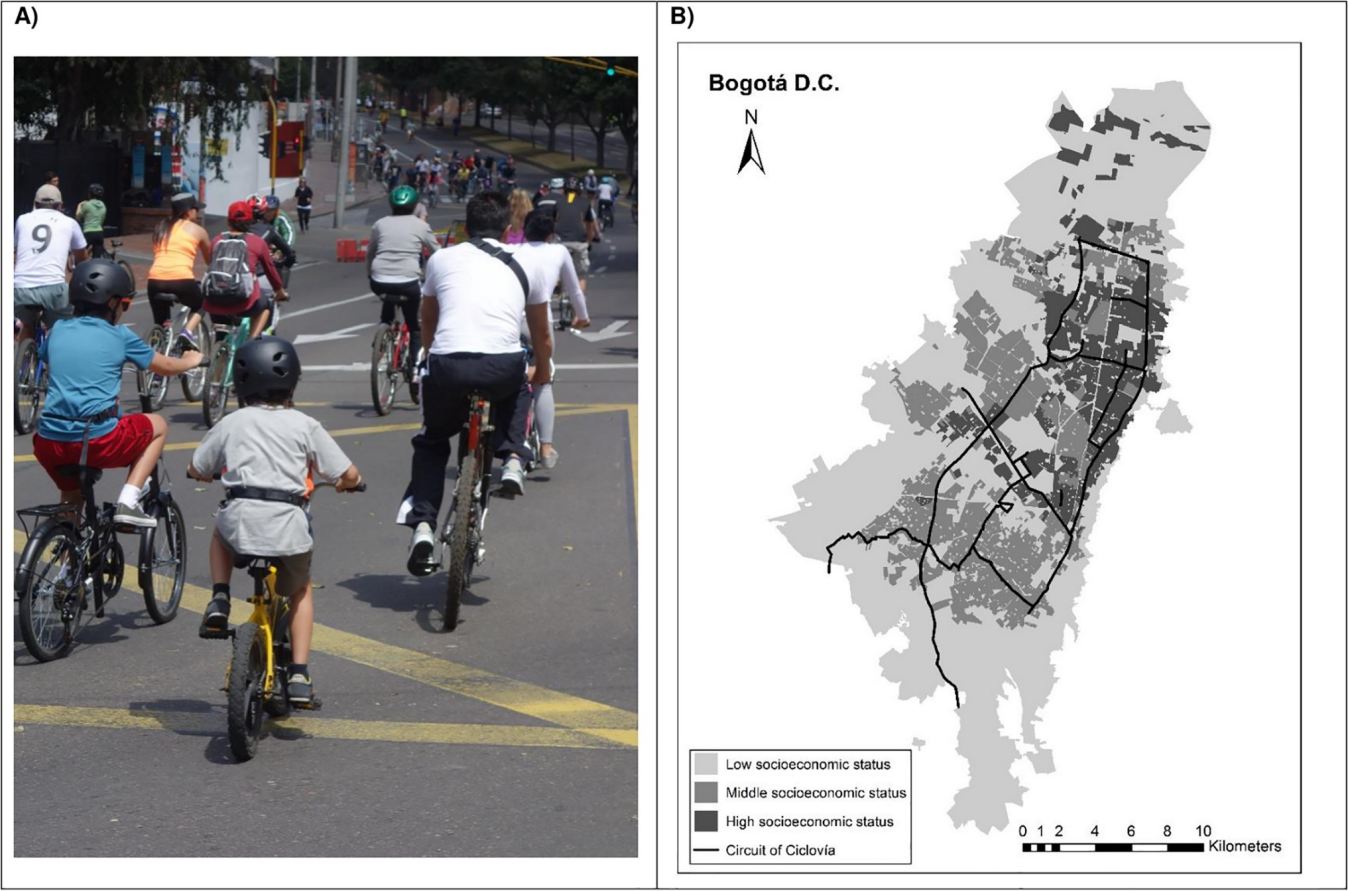
Ciclovía program of Bogotá. A) Photograph of the Ciclovía program with participation of children and their families. Photograph by Olga L. Sarmiento. B) Circuit of the Ciclovía of Bogotá crossing neighborhoods with different socioeconomic statuses. Image created by authors.

Sustainable interventions aimed at promoting active play among children and families require actions within and beyond the health sector to foster favorable social norms and provide equitable supportive environments and policies[10]. One promising program recommended in the Plan of Action for the Prevention of Obesity in Children and Adolescents developed by the Pan American Health Organization (PAHO) is the Ciclovía or Open Streets Program[11,12]. The Ciclovía is a multisectoral community intervention in which streets are temporarily closed to motorized transport to create a safe and free space exclusively for activities during leisure time[13] (Fig 1). This program provides a supportive environment for children and families to engage in activities in a safe setting, at least on weekends[14]. Currently, Ciclovías are implemented in at least 26 countries[15].

Previous studies have shown that adults who participate in the Ciclovía are more likely to meet the PA recommendations in their leisure time[14] and have a higher health-related quality of life and social capital scores[16] than their counterparts. In addition, by encouraging people to use the main streets, Ciclovía programs promote social inclusion, interaction and equity among citizens[11].

To date, no study has characterized children who use the Ciclovía or has assessed PA and SED among children who are frequent users of these programs. Therefore, the aim of this study is to assess the associations among PA, SED, body mass index (BMI), and Ciclovía participation among children aged 9 to 13 years.

## Methods

### Study setting

Bogotá is the capital city of Colombia and has over 7.8 million inhabitants[17]. The school-aged population of Bogotá for the year 2015 was almost 1.7 million children between the ages of 3 and 16 years old[18]. Regarding public spaces for leisure activities among children, Bogotá has 5,050 parks (3.9 m^2^/inhabitant)[19,20]. Regarding safety, in 2014, the crime indicators per 100,000 inhabitants were as follows: a robbery rate of 356.9[21], indiscriminate violence rate of 358.0[22], road traffic fatality rate of 8.0[21] and road traffic injury rate of 80.7[21]. Only 33% of the inhabitants of Bogotá perceive their neighborhoods as safe places[21]. In Bogotá, only 19.4% of children (6 to 12 years) and 13.4% of adolescents (13 to 17 years) meet the PA recommendations[23] and 21.0%. of children and adolescents are overweight or obese[24].

### The Ciclovía program

The Ciclovía of Bogotá was introduced in 1974 and since then has been considered an international model program for best practices[25]. Currently, Bogotá’s Ciclovía is the longest in the world[15] at more than 120 km (74.6 miles) in length. The number of participants ranges between 600,000 and 1,400,000 per event[11]. The Ciclovía occurs on Sundays and holidays, with approximately 72 events per year with a duration of 7 hours each (07 to 14 hours) [11]. This program has several complementary activities for children and families, such as “the bicycle school”, in which children and their families are taught how to ride bikes, and “the Recreovía program”, which offers PA classes for children and their families in parks close to the Ciclovía circuit[26].

### Study population

This article used data from two studies that were combined to increase the sample size (n = 1,107) [Colombia’s subsample of the International Study of Childhood Obesity, Lifestyle and Environment (ISCOLE)[27] and the sample of a school trial for the evaluation of the Active Recess Module (MARA) from the *Muévete Escolar* study of Bogotá]. These two studies followed the same methodology for assessment of the variables included in this analysis.

ISCOLE’s subsample included 919 students from 20 schools in Bogotá assessed in 2012. The schools were selected according to the following inclusion criteria: 1) located in urban areas; 2) following a January-December calendar; 3) elementary, middle and high school students enrolled in the same facility; 4) students included boys and girls; 5) a morning schedule; and 6) not serving a mainly disabled population (i.e., blind or deaf)[27]. The MARA subsample included 188 students from three schools assessed in 2013. The schools were selected according to the same criteria as the ISCOLE schools. Additionally, schools were selected in neighborhoods with streets used for the Ciclovía[28].

All students in the 4^th^ and 5^th^ grades from ISCOLE’s selected schools and all students in 5^th^ grade from the MARA selected schools were invited to participate. All participants signed an informed assent form and their parents or legal guardians signed an informed consent form before starting data collection. The student’s questionnaire was conducted face-to-face at the schools. The parent´s questionnaire was conducted through a face-to-face interview or by telephone. All questionnaires were conducted in a standardized manner by trained interviewers[27]. The Pennington Biomedical Research Center Institutional Review Board approved the ISCOLE protocol, and the Institutional Review Board of the Universidad de los Andes in Bogotá reviewed and approved all protocols and questionnaires for both studies.

### Physical activity and sedentary time

To objectively measure moderate-to-vigorous physical activity (MVPA) and SED among children, we used an ActiGraph GT3X+ accelerometer (ActiGraph LLC, Pensacola, FL, USA). Children were asked to use the accelerometer 24 hours per day for 7 days (plus an initial familiarization day and the morning of the final day) with an elasticized belt around the waist at the right mid-axillary line. The minimal amount of accelerometer data considered acceptable for the study was 4 days, including at least one weekend day, with 10 valid hours per day[27]. The Evenson equation[29] defined the MVPA and SED cut points. MVPA was defined as all activity ≥574 counts per 15 seconds, and SED was defined as all activity <25 counts per 15 seconds[30]. MVPA and SED were analyzed as continuous variables, and PA was dichotomized to meet the recommendations (<60 minutes per day vs ≥60 minutes per day)[31] and was compared between weekdays and weekends.

### Anthropometric measurements

Anthropometric measurements were obtained by trained staff. Height was measured using a Seca 213 portable stadiometer (Hamburg, Germany). The measurement was repeated, and a third measurement was obtained if the first two measures were separated by >0.5 cm. The mean of the two closest measurements was used for the analysis. Weight was measured using a portable Tanita SC-240 Body Composition Analyzer (Arlington Heights, IL, USA). Two measurements were obtained, and a third measurement was obtained if the first two measures were separated by >0.5 kg. The mean of the two closest measurements was used for the analysis[27]. The BMI (body mass (kg)/height^2^ (m^2^)) was calculated. The BMI z-score was computed based on reference values from the World Health Organization[32], and the participants were classified as either underweight (BMI z-score ≤-1 s.d.), normal weight (>-1 to <+1 s.d.), overweight (+1 to <+2 s.d.) or obese (BMI z-score ≥+2 s.d.).

### Sociodemographic variables

The sociodemographic variables included sex, age, highest level of parental education, neighborhood socioeconomic status (SES) and motorized vehicle availability in the household. We computed age from the date of birth to the observation date. Based on the highest educational level of the mother or father, we created the highest parental education variable and classified it into the following four categories: less than high school, uncompleted high school, high school diploma and university degree. In Bogotá, the SES is determined using the classification from the city Planning Department, which has 6 categories based on the physical characteristics of the household and neighborhood. SES category 1 corresponds to the poorest and category 6 to the wealthiest neighborhoods. In this study, the neighborhood SES was classified as either low (1–2), medium (3) or high (4, 5 and 6). Motorized vehicle availability was classified into two categories (no motorized vehicle and one or more motorized vehicles in the household).

### Environmental variables

#### Ciclovía perceptions

Both studies employed a self-reporting instrument designed to assess children’s behavior and Ciclovía participation patterns. Parents were asked the following questions: 1) “How often do you attend Ciclovía?”, 2) “How often does your child attend Ciclovía?”, 3) “What are the main activities your child usually performs when he/she attends Ciclovía?”, 4) “What is the average amount of time your child usually spends at Ciclovía?” and 5) “What is the average amount of time your child spends doing the previously mentioned activities during Ciclovía?” Additionally, for the MARA subsample, the children were asked: “With whom do you usually go to Ciclovía?” The frequency of attending Ciclovía for parents and children was coded into the following three categories: nonusers (never gone to Ciclovía), sporadic (<1 per month) and frequent (≥1 per month).

Ciclovía users were characterized according to the type of activity (cycling, skating, walking, jogging, riding a scooter, skateboarding, using a wheelchair and others), number of activities performed at Ciclovía, time spent at Ciclovía and time spent doing each activity. The mean time spent at Ciclovía and the time doing activities at Ciclovía were classified into <60 minutes and ≥60 minutes (the PA daily recommendations).

#### Safety perception

To evaluate the perception of crime, an adapted subscale based on the Neighborhood Environment Walkability Scale was used[33]. The scale comprised the following 5 questions: 1) “Is the criminality rate high?”, 2) “Are you afraid an unknown person will injure or kidnap your child in the neighborhood streets?”, 3) “Are you afraid an unknown person will injure or kidnap your child in your garden, the entrance of your house or a common area?”, 4) “Are you afraid an unknown person will injure or kidnap your child in a local park?” and 5) “Are you afraid a malicious person (adult or child) may injure or kidnap your child inside your neighborhood?”. These items were assessed using a 4-point Likert scale, and the mean was obtained. We used the Loess (locally weighted smoothing) method, which creates a smooth line, to assess the shape of the relationship between safety perception and attendance at the Ciclovía[34].

#### Opportunities to practice physical activity

The distance to parks and the Ciclovía circuit were estimated using ArcGIS 10.2 (ESRI Inc., Redlands, CA, USA). The children’s home addresses were reported by parents in the demographic questionnaire. The home, park and Ciclovía circuit addresses were geo-referenced. If the parents did not provide a complete address, the closest street intersection was used. To estimate the distance between home and parks or the Ciclovía circuit, we assumed that the children took the shortest route via the street network. The cut point of distance for parks and Ciclovía was defined based on the shape of the Loess curve[34].

### Statistical analysis

Associations among MVPA, SED and BMI with participation in the Ciclovía were estimated using a multilevel linear mixed model (SAS PROC MIXED). Associations between meeting the PA daily recommendations, being overweight and Ciclovía participation were estimated in terms of odds ratios using generalized linear mixed models (SAS PROC GLIMMIX). Schools were considered random effects. The denominator degrees of freedom for statistical tests pertaining to fixed effects were calculated using the Kenward and Roger approximation. The models included sex, age, highest level of parental education, SES, availability of motorized vehicles and crime perception as covariates. These analyses were conducted with SAS 9.4 (SAS, Cary, NC, USA, 2007).

We conducted an additional analysis to assess differences between frequent vs sporadic and non-Ciclovía users in the hourly distribution of MVPA and SED on Sundays, Saturdays and weekdays. These comparisons were conducted using the generalized additive model (GAM) function of the MGCV package in R version 3.0.1 (R Foundation for Statistical Computing) with a smooth covariate function (time of day) for each group. The GAM was fitted according to the data using a Poisson distribution with a log-link function, and thin-plate regression splines were used to estimate a smooth function.

## Results

### Sociodemographic characteristics

Of the 1,107 children enrolled in this study, 923 children remained in the analytic dataset after excluding participants who did not have valid accelerometry data for Sundays (n = 182) and the SES (n = 2). The participants who were excluded from this analysis did not have differences in sociodemographic variables.

Approximately half of the children were boys aged 10.1±0.7 years. Most participants had parents with at least a high school diploma (68.3%), lived in low SES neighborhoods (77.5%), did not have a motorized vehicle (cars, trucks or motorcycles) in their household (75.7%) and considered their neighborhoods unsafe places (67.2%) (Table 1).

**Table 1:** Sociodemographic characteristics of participants of analytical sample of the study.

Characteristics	N (%)	P-value
Total	923	
Sex		
Male	466 (50.5)	0.767
Female	457 (49.5)	
Age (years)		
9 to 10	706 (76.49)	<0.001
11 to 13	217 (23.51)	
Highest parental education		
Less than secondary/high school	72 (11.9)	<0.001
Some secondary/did not complete high school	135 (22.2)	
High school diploma	223 (36.7)	
Technical, undergraduate or postgraduate degree	177 (29.2)	
Socioeconomic status		
Low (1–2)	717 (77.7)	<0.001
Medium (3)	162 (17.5)	
High (4–6)	44 (4.8)	
Availability of motorized vehicles in household		
None	700 (75.8)	<0.001
≥ 1	223 (24.2)	
Neighborhood crime perception^a^		
Safe	303 (32.8)	<0.001
Unsafe	620 (67.2)	
Distance to parks^a^		
≤ 120 m	618 (67.0)	<0.001
> 120 m	305 (33.0)	
Distance to Ciclovías^a^		
≤ 1,600 m	519 (56.2)	<0.001
> 1,600 m	404 (43.8)	

^a^ Cut point determined by the Loess curve

### Ciclovía participation

In this study, 47% of the parents reported that their child had participated in Ciclovía within the last year, and 34% reported frequent participation. Participation was similar by sex (male 49.5%, female 50.5%, p = 0.14), and 93% of the children lived in low-to-middle-income neighborhoods. Frequent Ciclovía users attended once per month (45.1%), twice per month (27.3%), thrice per month (6.0%) and four or more times per month (21.6%). The main activities performed at Ciclovía were cycling (66.7%), walking (36.3%) and skating (16.9%). The majority of participants engaged in only one activity (52.6%), spent at least 60 minutes at Ciclovía (97.0%), and reported being active for ≥60 minutes at Ciclovía (88.9%) (Table 2). Frequent Ciclovía users were more likely to perceive the neighborhood as safe (40.6% vs 28.8%) and live nearer to the Ciclovía (64.4% vs 52.0%). Furthermore, in the MARA subsample, Ciclovía users reported participating in the program with their parents (75.7%), brothers and sisters (55.3%), other relatives (23.3%) and friends (4.9%).

**Table 2:** Characteristics of the Ciclovía users.

Variables	Sporadic Ciclovía users^a^, N (%)	Frequent Ciclovía users^b^, N (%)
Frequency of attendance at Ciclovía		
At least one time in the last year	117 (100)	-
One time per month	-	142 (45.1)
Two times per month	-	86 (27.3)
Three times per month	-	19 (6.0)
Four or more times per month	-	68 (21.6)
Type of activity performed^c^		
Bicycling	66 (56.4)	222 (70.5)
Walking	45 (38.5)	112 (35.6)
Skating	11 (9.4)	62 (19.7)
Jogging	6 (5.1)	23 (7.3)
Skateboarding	7 (6.0)	13 (4.1)
Scooter riding	0 (0.0)	2 (0.6)
Wheelchair use	1 (0.8)	3 (1.0)
Other	1 (0.9)	3 (1.0)
Number of activities at Ciclovía		
One	3 (2.6)	224 (71.1)
Two	94 (80.3)	60 (19.0)
Three or more	20 (17.1)	31 (9.8)
Time spent at Ciclovía		
<60 minutes	2 (1.7)	11 (3.5)
≥60 minutes	115 (98.3)	304 (96.5)
Time spent at Ciclovía doing physical activity		
<60 minutes	9 (7.7)	39 (12.4)
≥60 minutes	108 (92.3)	276 (87.6)

^a^ Participation in Ciclovía <1 time per month

^b^ Participation in Ciclovía ≥1 times per month

^c^ Includes ≥ 1 activity per participant

### Physical activity

On weekdays, 61.8% of children met the daily PA recommendations compared with 41.6% on Sundays. On weekdays, frequent Ciclovía users did not differ from sporadic and non-Ciclovía users in the minutes of MVPA (72 vs 69 min; difference = 2.7; CI = -0.4 to 5.9; p = 0.09). On Sundays, the frequent Ciclovía users had higher MVPAs than the sporadic and non-Ciclovía users (66 vs 59 min; difference = 6.3; CI = 1.2–11.4; p = 0.015) (Table 3).

**Table 3:** Adjusted mean minutes of weekday and Sunday MVPA, SED and adiposity by Ciclovía participation.

Variable	Mean	Difference	s.e.m^f^	Lower 95% CI	Upper 95% CI	P-value
MVPA^a^, min per day (weekday)						
Frequent Ciclovía users^b^	71.65	2.71	1.60	-0.43	5.86	0.09
Sporadic and non Ciclovía users^c^	68.94	-	-	-	-	-
SED^d^, min per day (weekday)						
Frequent Ciclovía users^b^	515.32	-6.01	4.591	-15.02	3.00	0.19
Sporadic and non Ciclovía users^c^	521.33	-	-	-	-	-
MVPA^a^, min per day (Sunday)						
Frequent Ciclovía users^b^	65.54	6.32	2.60	1.22	11.41	0.02
Sporadic and non Ciclovía users^c^	59.23	-	-	-	-	-
SED^d^, min per day (Sunday)						
Frequent Ciclovía users^b^	437.69	-18.79	6.68	-31.90	-5.6825	0.005
Sporadic and non Ciclovía users^c^	456.48	-	-	-	-	-
Body Mass Index^e^						
Frequent Ciclovía users^b^	0.30	0.17	0.07	0.03	0.30	0.01
Sporadic and non Ciclovía users^c^	0.13	-	-	-	-	-

Models were adjusted by age, sex, socioeconomic status, household motorized vehicles, highest parental education, criminality and distance to Ciclovía and parks

^a^ Moderate-to-vigorous physical activity data shown as adjusted mean

^b^ Attendance to Ciclovía ≥1 times per month

^c^ Non Ciclovía users and attendance to Ciclovía <1 time per month

^d^ Sedentary time data shown as adjusted mean

^e^ Body Mass Index z-score according to WHO reference data

^f^ Standard error for mean

Compliance with PA guidelines on Sundays was associated with being a frequent Ciclovía user (OR = 1.27; CI = 0.94–1.72), a boy (OR = 1.92; CI = 1.45–2.55), living in low (OR = 5.72; CI = 1.98–16.53) and middle SES neighborhoods (OR = 2.86; CI = 0.99–8.16) and living in households without motorized vehicles (OR = 1.36; CI = 0.96–1.92).

### Sedentary time

On weekdays, frequent Ciclovía users did not differ from sporadic and non-Ciclovía users in SED (515.32 vs 521.33 min; difference = -6.01; CI = -15.02 to 3.00; p = 0.19). On Sundays, frequent Ciclovía users had lower SED than sporadic and non-Ciclovía users (438 vs 456 min; difference = -18.8; CI = -31.9 to-5.7; p = 0.005) (Table 3).

**Fig 2:**
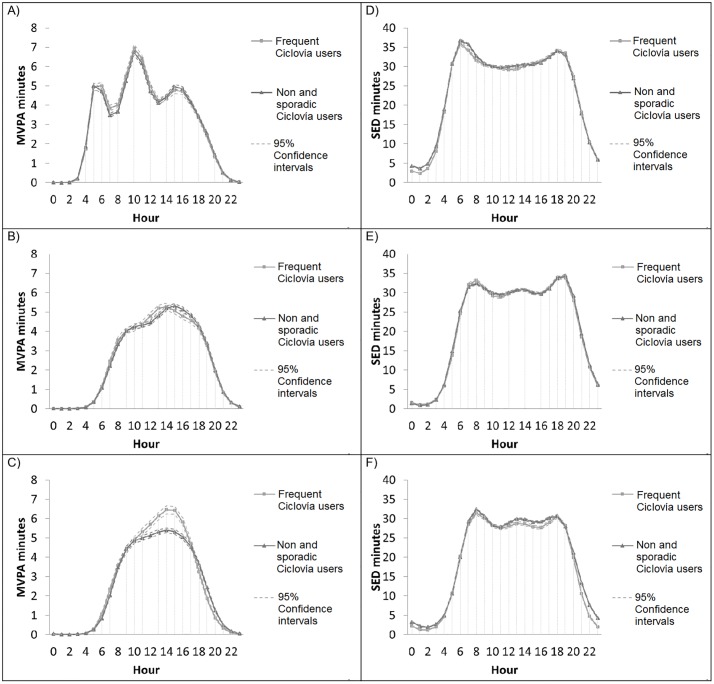
Predictive minutes of moderate-to-vigorous physical activity (MVPA) and sedentary time (SED) for frequent users vs sporadic and nonusers of Ciclovía. A) MVPA minutes on weekdays, B) MVPA minutes on Saturday, C) MVPA minutes on Sunday, D) SED minutes on weekdays, E) SED minutes on Saturday, F) SED minutes on Sunday.

MVPA and SED on weekdays and Saturdays were similar between the frequent and sporadic and non-Ciclovía users (Fig 2). Frequent Ciclovía users on Sundays recorded higher mean minutes of MVPA between the hours of 12:00 and 16:00 and slightly lower mean minutes of SED between 12:00 and 18:00.

### Weight status

Most participants were classified in the normal weight category (65.3%), followed by overweight (17.6%), underweight (11.6%) and obese (5.5%). Frequent Ciclovía users were more likely to be overweight and obese (28.3% vs 20.4%, p = 0.01) and to have higher mean BMI z-scores than their counterparts (0.30 vs 0.13; difference = 0.17; CI = 0.03–0.30; p = 0.01) (Table 3).

A high BMI was associated with being a frequent Ciclovía user (OR = 1.29; CI = 1.04–1.60), a boy (OR = 1.26; CI = 1.04–1.53) and living in a high SES neighborhood (OR = 1.20; CI = 0.70–2.07).

## Discussion

We believe that this study is the first to examine the associations between frequent Ciclovía participation with PA, SED and being overweight among children. Our findings showed that frequent Ciclovía users recorded an average of 6 more minutes of MVPA on Sundays than sporadic and non-Ciclovía users. Additionally, frequent Ciclovía users recorded 19 minutes less SED on Sundays. Frequent Ciclovía users also had higher BMI z-scores. Reflecting the inclusive nature of the Ciclovía program and its potential to decrease SES gaps, we did not find differences in participation by sex, and low-to-middle SES children were more likely to participate in the program than those from families with high incomes.

**Fig 3:**
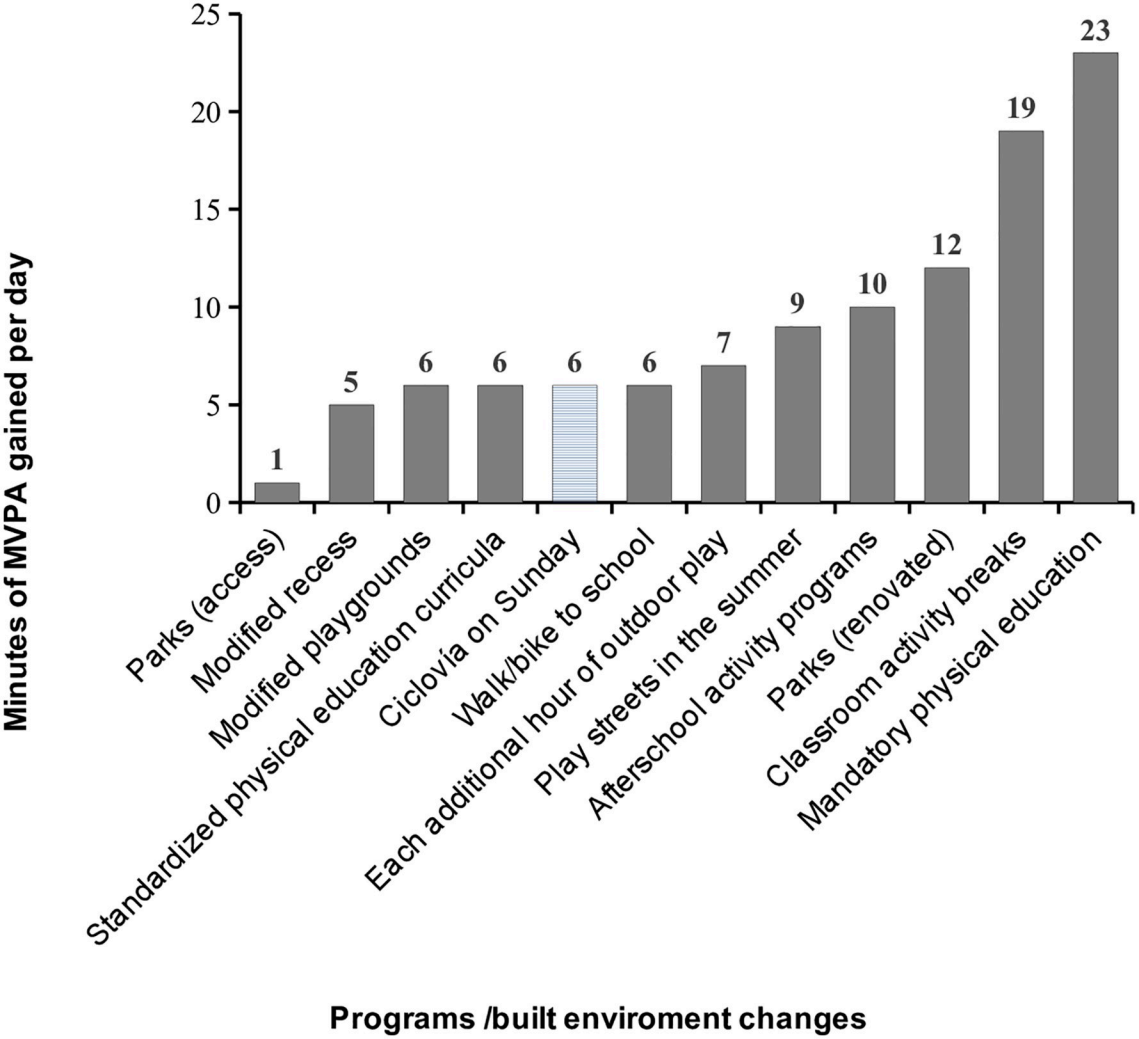
Minutes of moderate-to-vigorous physical activity gained per day resulting from programs and built environment changes. Note: This figure is an adaptation of data presented by Bassett et al[38], D’Haese et al[40] and Denstel et al[39]. MVPA: moderate-to-vigorous physical activity.

Previous studies have shown that children are more likely to meet PA recommendations on weekdays than on weekends[35–37]. In this context, the Ciclovía has the potential to increase outdoor MVPA time on weekends while encouraging shared time for families. Furthermore, the amount of daily MVPA time provided by the Ciclovía is comparable to the time reported for modified playgrounds, standardized playgrounds[38] and through active school transport[39] (Fig 3).

These results are consistent with those of other studies of Play Streets (temporarily closing streets to traffic so children and parents can play outside[41]). Children in Belgium recorded higher MVPA and lower SED during a Play Street intervention during the summer[40]. Likewise, children in Chile recorded higher total daily step counts during the afterschool “*Juega en tu Barrio*” Play Street intervention among low income neighborhoods[42]. Furthermore, these interventions have the potential to increase outdoor time for children. In fact, children prefer outdoor play because it provides the opportunity to run and move[43,44]. In addition, play provides benefits that extend beyond physical health, including social and emotional well-being[45,46] and cognitive development[47].

Frequent Ciclovía participants had lower mean minutes of SED. Generally, interventions to reduce SED among school-age children do not have evidence of effectiveness[48]. The Ciclovía is a program that provides a supportive environment with components of social support from family and friends. These elements have been effective in interventions to reduce SED among preschool children[49] and should be evaluated in longitudinal studies that include school-aged children participating in the Ciclovía.

Despite the significant relationships with more PA and less SED, frequent Ciclovía participation was associated positively with the BMI z-scores. A possible explanation for this association could be that the main or only activity for these children occurred in the Ciclovía. In fact, no differences in PA and SED were found during the weekdays. Therefore, the amount of MVPA occurred only on Sundays, which might not be sufficient to prevent being overweight.

Neighborhood security, traffic perceptions and fear of crime among parents may lead to closer supervision of their children[50] and limiting outdoor recreational opportunities[42,51]. Our study found that frequent users of the Ciclovía were more likely to perceive the neighborhood as safe. These results are consistent with studies evaluating Play Streets[42] and active free play[52]. In the “*Juega en tu Barrio”* intervention, parents reported an increased perception of safety after the road was closed to traffic[42]. Programs such as Play Streets[42] and the Ciclovía offer an inclusive, safer and innovative use of existing public spaces for recreation.

This study has important national and international policy implications. For Colombia, 34% of children and adolescents live in poverty[53], which underscores the need for safe recreational and public spaces for play and having fun[53]. Our results also provide evidence for 1) the continuity and expansion of complementary activities for children within the Ciclovía, 2) the expansion of this program to the country through the Active and Healthy Streets (Vías Activas y Saludables) program from the ministry of sports (Coldeportes) and 3) its recommendation within Colombia’s national obesity law[54]. Internationally, the Ciclovía program is recognized as a model program by the PAHO[12], the National Collaborative on Childhood Obesity Research[55] and the World Bank Group[56] and has been proven to be feasible for implementation in low-, middle- and high-income countries[15].

### Strengths and limitations

This study uses objective measures of MVPA and SED with a sample of almost 1,000 children in the world’s longest Ciclovía. Nonetheless, our findings should be interpreted with caution. First, using waist-mounted accelerometers to measure PA in this study can underestimate activity during cycling (the preferred activity during the Ciclovía)[57]. Given that we found significantly higher MVPA in frequent Ciclovía participants, our results may be conservative. Second, because this study is cross-sectional, we are unable to determine the direction of causality. Third, parents reported only the participation of their children in the Ciclovía without specific details of the time for each activity. Future studies should include a daily record of activities combined with GPS and GIS, which would enhance the accuracy of Ciclovía participation. Lastly, the small number of Ciclovía users every week did not allow us to assess this independent category.

In conclusion, the Ciclovía is an inclusive program that has the potential to increase outdoor time, promote PA and decrease SED among children. An increase in public spaces for recreation through the Ciclovía, which is expanding worldwide, could allow children and families to gain access to free recreational spaces.

## Supporting information

S1 File ISCOLE demographic and family health questionnaire for parents. (PDF)

S2 File ISCOLE diet and lifestyle questionnaire for children. (PDF)

S3 File ISCOLE Anthropometric Data Collection Form. (PDF)

S4 File ISCOLE demographic and family health questionnaire for parents (Spanish). (DOCX)

S5 File ISCOLE diet and lifestyle questionnaire for children (Spanish). (PDF)

S6 File MARA demographic and family health questionnaire for parents. (DOCX)

S7 File MARA diet and lifestyle questionnaire for children. (DOCX)
