# Supplementary material for: Active streets for children: The case of the Bogotá Ciclovía
Source: PLoS One. 2019 May 15;14(5):e0207791. doi: 10.1371/journal.pone.0207791 (PMC6519789; doi:10.1371/journal.pone.0207791)
Supplement: S1 File — (PDF) [file pone.0207791.s001.pdf]

Participant ID
(attach label here)

Technician Initials ☐☐☐

Date ☐☐ / ☐☐☐ / ☐☐☐☐
Day Month Year

APPENDIX E: ISCOLE Demographic and Family Health Questionnaire

A. GENERAL INFORMATION

Child's Name:

Last First Middle

Name of Child's School:

Parent's or Guardian's Name:

Last First Middle

Home Address:

Street Address Apt. # Town or City State Postal/Zip Code

Nearest Cross-Street to Home:

Phone Number: (   ) E-Mail:

Area Code

How long have you lived at the current address? _________years and _________months

B. DEMOGRAPHICS OF CHILD

Birth date _____/_____/_____
dd/mmm/yyyy
Example: 02/Jun/2011

Age _____years

Gender: ☐ Male ☐ Female

Ethnicity:

- ☐ White
- ☐ African American
- ☐ Asian
- ☐ American Indian, Aleutian, Alaska native or Eskimo
- ☐ Pacific Islander
- ☐ Don't know
- ☐ Other ___________________

Are you of Hispanic origin? ☐ Yes ☐ No

ISCOLE QUALITY CONTROL (QC) and DATA ENTRY:

QC Staff Initials: ____________________________ Date: ______ / ________ / _______

Data Entry Staff Initials: ______________________ Date: ______ /________ / _______

Participant ID (attach label here)	Technician Initials ☐☐☐
	Date ☐☐ / ☐☐☐ / ☐☐☐☐<br>Day Month Year

In what country was the child born? ___________________

How many biological brothers and sisters does the child have? _______________

What are their ages? _____yrs _____yrs _____yrs _____yrs _____yrs
_____yrs _____yrs _____yrs _____yrs _____yrs

C. HEALTH HISTORY OF CHILD

1. Birth Weight: _______kg OR _______lbs &_____oz Birth Length: ____cm OR ____inches

2. Length of Pregnancy: _______weeks OR ____months

3. Did mother develop gestational diabetes during pregnancy with **THIS** child? ☐ Yes No ☐

4. Fed breast milk? ☐ Yes ☐ No If No, please skip to question 5.

Age when **COMPLETELY** stopped being fed breast milk: _____months

Age when **FIRST** fed formula: _____months

5. Age when **COMPLETELY** stopped drinking formula: _____months

C. FAMILY DEMOGRAPHICS AND HEALTH

6. What is the marital status of the child's parents?

- ☐ Married
- ☐ Divorced or separated
- ☐ Never married
- ☐ Widowed parent

7. How many people live in your household (at this address)? ____

7a. Who lives with the child **at this address** (check all that apply)?

☐ Biological Mother	☐ Brother(s) or Sister(s)
☐ Biological Father	☐ Grandparent(s)
☐ Adoptive Mother	☐ Other Relative(s)
☐ Adoptive Father	☐ Friend(s)
☐ Step Mother	☐ Legal Guardian(s)
☐ Step Father	☐ OTHER

ISCOLE QUALITY CONTROL (QC) and DATA ENTRY:

QC Staff Initials: ____________________________ Date: ______ / ________ / _______

Data Entry Staff Initials: ______________________ Date: ______ /________ / _______

Participant ID
(attach label here)

Technician Initials ☐☐☐

Date ☐☐ / ☐☐☐ / ☐☐☐☐
Day Month Year

8. What is the **COMBINED** annual income for your household (before taxes)?

- ☐ Less than $10,000
- ☐ $10,000 - $29,999
- ☐ $30,000 - $49,999
- ☐ $50,000 - $69,999
- ☐ $70,000 - $89,999
- ☐ $90,000 - $109,999
- ☐ $110,000 - $139,999
- ☐ $140,000 and above

9. How many functioning motorized vehicles (car, truck, motorcycle, moped, etc.) are available for use at your house?

- ☐ 0
- ☐ 1
- ☐ 2
- ☐ 3
- ☐ 4
- ☐ 5 or more

10. How many television sets are in your household?

- ☐ 0
- ☐ 1
- ☐ 2
- ☐ 3
- ☐ 4
- ☐ 5 or more

11. What best describes your type of television service for the **primary** television in the house?

- ☐ No television
- ☐ Antenna only
- ☐ Basic cable
- ☐ Cable + premium channel(s)
- ☐ Satellite dish
- ☐ Other
- ☐ Don't know

ISCOLE QUALITY CONTROL (QC) and DATA ENTRY:

QC Staff Initials: ____________________________ Date: ______ / ________ / _______

Data Entry Staff Initials: ______________________ Date: ______ /________ / _______

Participant ID
(attach label here)

Technician Initials ☐☐☐

Date ☐☐ / ☐☐☐ / ☐☐☐☐
Day Month Year

12. What best describes your type of internet service?

- ☐ No internet access
- ☐ Dial-up modem
- ☐ DSL modem
- ☐ Cable modem
- ☐ Other
- ☐ Don't know

13. What is the **MOTHER'S** highest level of education completed?

- ☐ Less than high school
- ☐ Some high school
- ☐ High school diploma/GED
- ☐ Associate's degree or 1-3 years of college
- ☐ Bachelor's degree
- ☐ Graduate/professional degree

14. How many hours per week does the **MOTHER** work <u>outside</u> the home?

- ☐ None
- ☐ Less than 15 hours/week
- ☐ 15-35 hours per week
- ☐ Full time (36+ hours per week)

15. What is the **FATHER'S** highest level of education completed?

- ☐ Less than high school
- ☐ Some high school
- ☐ High school diploma/GED
- ☐ Associate's degree or 1-3 years of college
- ☐ Bachelor's degree
- ☐ Graduate/professional degree

16. How many hours per week does the **FATHER** work <u>outside</u> the home?

- ☐ None
- ☐ Less than 15 hours/week
- ☐ 15-35 hours per week
- ☐ Full time (36+ hours per week)

ISCOLE QUALITY CONTROL (QC) and DATA ENTRY:

QC Staff Initials: ____________________________ Date: ______ / ________ / _______

Data Entry Staff Initials: ______________________ Date: ______ /________ / _______

Participant ID
(attach label here)

Technician Initials ☐☐☐

Date ☐☐ / ☐☐☐ / ☐☐☐☐
Day Month Year

17. Is this child adopted? ☐ Yes ☐ No

18. Please answer the following questions with regard to the child's **BIOLOGICAL MOTHER**:

Current height: _____cm **or** _____feet and _______inches Current weight: _______kg **or** _______lbs

Current Age: _____ years

Age at child's birth: ____ years

☐ Biological Mother's information cannot be estimated or is not known

19. Please answer the following questions with regard to the child's **BIOLOGICAL FATHER:**

Current height: _____cm **or** _____feet and _______inches Current weight: _______kg **or** _______lbs

Current age: _____ years

☐ Biological Father's information cannot be estimated or is not known

ISCOLE QUALITY CONTROL (QC) and DATA ENTRY:

QC Staff Initials: ____________________________ Date: ______ / ________ / _______

Data Entry Staff Initials: ______________________ Date: ______ /________ / _______
