# Supplementary material for: Active streets for children: The case of the Bogotá Ciclovía
Source: PLoS One. 2019 May 15;14(5):e0207791. doi: 10.1371/journal.pone.0207791 (PMC6519789; doi:10.1371/journal.pone.0207791)
Supplement: S2 File — (PDF) [file pone.0207791.s002.pdf]

Participant ID (attach label here)	Technician Initials ☐☐☐<br>Date ☐☐ / ☐☐☐ / ☐☐☐☐<br>Day Month Year

Appendix M: ISCOLE Diet and Lifestyle Questionnaire

Please read every question carefully. What answer comes to your mind first?
Choose the box that fits your answer best and fill it in.

Remember: This is not a test so there are no wrong answers. It is important that you answer all the questions and that we can see your marks clearly.

You do not have to show your answers to anybody. Also, nobody who knows you will look at your questionnaire once you have finished it.

For the questions on this page, please tell about what you did *last week*.

1. On a school day, how many hours did you watch TV?

   ☐ I did not watch TV on school days ☐ < 1 hour ☐ 1 hour ☐ 2 hours ☐ 3 hours ☐ 4 hours ☐ 5 or more hours

2. On a school day, how many hours did you play video or computer games or use a computer for something that was not school work?

   ☐ I did not play video/computer games or use a computer other than for school work on school days ☐ < 1 hour ☐ 1 hour ☐ 2 hours ☐ 3 hours ☐ 4 hours ☐ 5 or more hours

3. On a school day how much time did you spend outside **before** school?

   ☐ < 1 hour ☐ 1 hour ☐ 2 hours ☐ 3 hours ☐ 4 hours ☐ 5 or more hours

4. On a school day how much time did you spend outside **after** school before bedtime?

   ☐ < 1 hour ☐ 1 hour ☐ 2 hours ☐ 3 hours ☐ 4 hours ☐ 5 or more hours

5. On a weekend day, how many hours did you watch TV?

   ☐ I did not watch TV on weekend days ☐ < 1 hour ☐ 1 hour ☐ 2 hours ☐ 3 hours ☐ 4 hours ☐ 5 or more hours

6. On a weekend day, how many hours did you play video or computer games or use a computer for something that was not school work?

   ☐ I did not play video/computer games or use a computer other than for school work on the weekend ☐ < 1 hour ☐ 1 hour ☐ 2 hours ☐ 3 hours ☐ 4 hours ☐ 5 or more hours

7. On a weekend day, how much time did you spend outside?

ISCOLE QUALITY CONTROL (QC) and DATA ENTRY:

QC Staff Initials: ____________________________ Date: ______ / ________ / _______

Data Entry Staff Initials: ______________________ Date: ______ /________ / _______

Participant ID (attach label here)	Technician Initials ☐☐☐ Date ☐☐ / ☐☐☐ / ☐☐☐☐ (Day / Month / Year)

☐ < 1 hour ☐ 1 hour ☐ 2 hours ☐ 3 hours ☐ 4 hours ☐ 5 or more hours

8. In the last week you were in school, on how many days did you go to physical education (PE) classes?

☐ 0 days ☐ 1 day ☐ 2 days ☐ 3 days ☐ 4 days ☐ 5 days

9. In the last week you were in school, the **MAIN** part of your journey to school was by:

☐ walking
☐ bicycle, roller-blade, skateboard or scooter
☐ bus, train, tram, underground or boat
☐ car, motorcycle or moped
☐ other _______________

10. In the last week you were in school, **HOW LONG** did it take you to travel to school?

☐ < 5 minutes ☐ 5 - 15 minutes ☐ 16 - 30 minutes ☐ 31 minutes to 1 hour ☐ >1 hour

11. During the past year (12 months), did you do any of these activities? (Check all that apply)

☐ sports teams ☐ dance / martial arts class ☐ art / music class ☐ none of these

12. During the past week (7 days), on how many days were you physically active for a total of at least 60 minutes per day? (all the time you spent in activities that increased your heart rate and made you breathe hard)

☐ 0 days ☐ 1 day ☐ 2 days ☐ 3 days ☐ 4 days ☐ 5 days ☐ 6 days ☐ 7 days

Please tick the box that most sounds like you:

	Disagree a Lot → 1	2	3	4	5 Agree a Lot
13. I can be physically active during my free time on most days.	☐	☐	☐	☐	☐
14. I can ask my parent or other adult to do physically active things with me.	☐	☐	☐	☐	☐
15. I can be physically active during my free time on most days even if I could watch TV or play video games instead.	☐	☐	☐	☐	☐
16. I can be physically active during my free time on most days even if it is very hot or cold outside.	☐	☐	☐	☐	☐
17. I can ask my best friend to be physically active with me during my free time on most days.	☐	☐	☐	☐	☐
18. I can be physically active during my free time on most days even if I have to stay at home.	☐	☐	☐	☐	☐
19. I have the coordination I need to be physically active during my free time on most days.	☐	☐	☐	☐	☐
20. I can be physically active during my free time on most days no matter how busy my day is.	☐	☐	☐	☐	☐

ISCOLE QUALITY CONTROL (QC) and DATA ENTRY:

QC Staff Initials: _____________________________ Date: ______ / ________ / _______

Data Entry Staff Initials: _______________________ Date: ______ /________ / _______

Participant ID (attach label here)	Technician Initials ☐☐☐<br>Date ☐☐ / ☐☐☐ / ☐☐☐☐<br>Day / Month / Year

There are lots of reasons why people take part in physical activity. Please tick the box to show how much each of the reasons below is true for you:

	never true for me	a little bit true for me	sometimes true for me	true for me	very true for me
21. I take part in exercise because other people say I should	☐	☐	☐	☐	☐
22. It's important to me to exercise regularly	☐	☐	☐	☐	☐
23. I can't see why I should bother exercising	☐	☐	☐	☐	☐
24. I feel like a failure when I haven't exercised in a while	☐	☐	☐	☐	☐
25. I find exercise a pleasurable activity	☐	☐	☐	☐	☐

26. During the past week, what time have you usually turned out the light and gone to sleep on school days?

☐☐:☐☐ AM / PM (circle AM or PM)

27. During the past week, at what time have you usually woken up in the morning on school days**?**

☐☐:☐☐ AM / PM (circle AM or PM)

28. During the past week, what time have you usually turned out the light and gone to sleep on weekend days?

☐☐:☐☐ AM / PM (circle AM or PM)

29. During the past week, at what time have you usually woken up in the morning on weekend days?

☐☐:☐☐ AM / PM (circle AM or PM)

30. During the past week, how would you rate your sleep **quality** overall (how **well** you sleep)?

☐ very good ☐ fairly good ☐ fairly bad ☐ very bad

31. During the past week, how would you rate your sleep **quantity** overall (how **much** you sleep)?

☐ very good ☐ fairly good ☐ fairly bad ☐ very bad

32. Do you have a television in your bedroom?

ISCOLE QUALITY CONTROL (QC) and DATA ENTRY:

QC Staff Initials: ____________________________ Date: ______ / ________ / _______

Data Entry Staff Initials: ______________________ Date: ______ /________ / _______

Participant ID (attach label here)
☐ Yes ☐ No

**Technician Initials**	☐☐☐
**Date**	☐☐ / ☐☐☐ / ☐☐☐☐
	Day Month Year

33. How many times do you usually eat . . . ? (Please mark only one box for each line)

	Never	Less than once a week	Once a week	2-4 days a week	5-6 days a week	Once a day, every day	Every day, more than once
Fruits	☐	☐	☐	☐	☐	☐	☐
Vegetables	☐	☐	☐	☐	☐	☐	☐
Sweets (candy/chocolate)	☐	☐	☐	☐	☐	☐	☐
Regular cola or soft drinks that contain sugar	☐	☐	☐	☐	☐	☐	☐
Cake, pastries, or donuts	☐	☐	☐	☐	☐	☐	☐
Diet cola or diet soft drinks	☐	☐	☐	☐	☐	☐	☐
Potato chips	☐	☐	☐	☐	☐	☐	☐
French fries	☐	☐	☐	☐	☐	☐	☐
Dark green vegetables (broccoli, spinach, etc.)	☐	☐	☐	☐	☐	☐	☐
Orange vegetables (carrots, squash, sweet potato, etc.)	☐	☐	☐	☐	☐	☐	☐
Fruit juice	☐	☐	☐	☐	☐	☐	☐
Low fat milk (1%,2%, skim)	☐	☐	☐	☐	☐	☐	☐
Whole milk (homogenized)	☐	☐	☐	☐	☐	☐	☐
Cheese	☐	☐	☐	☐	☐	☐	☐
Other milk products (yogurt, chocolate milk, pudding, etc.)	☐	☐	☐	☐	☐	☐	☐
Whole grain bread or cereal (oatmeal, muesli, etc.)	☐	☐	☐	☐	☐	☐	☐
Meat alternatives (beans, lentils, tofu, eggs, peanut butter, etc.)	☐	☐	☐	☐	☐	☐	☐
Energy drinks (Red Bull, Rock Star, Guru, etc.)	☐	☐	☐	☐	☐	☐	☐
Sports drinks (Gatorade, Powerade, etc.)	☐	☐	☐	☐	☐	☐	☐
Fish	☐	☐	☐	☐	☐	☐	☐
Ice cream	☐	☐	☐	☐	☐	☐	☐

ISCOLE QUALITY CONTROL (QC) and DATA ENTRY:

QC Staff Initials: ______________________________ Date: ______ / ________ / _______

Data Entry Staff Initials: _______________________ Date: ______ /________ / _______

Participant ID (attach label here)	Technician Initials ☐☐☐
	Date ☐☐ / ☐☐☐ / ☐☐☐☐ (Day / Month / Year)

Fried food such as chicken wings, chicken fingers, etc.	☐	☐	☐	☐	☐	☐	☐
Fast foods such as pizza, hamburgers, etc.	☐	☐	☐	☐	☐	☐	☐

34. How many times do you usually eat the following food items **while watching television**?

	Never	Less than once a week	Once a week	2-4 days a week	5-6 days a week	Once a day, every day	Every day, more than once
Potato chips or peanuts	☐	☐	☐	☐	☐	☐	☐
Fried food such as chicken wings, chicken fingers, french fries, etc.	☐	☐	☐	☐	☐	☐	☐
Cookies, biscuits, chocolate or candy bars	☐	☐	☐	☐	☐	☐	☐
Ice cream	☐	☐	☐	☐	☐	☐	☐
Fast foods such as pizza, hamburgers, etc.	☐	☐	☐	☐	☐	☐	☐
Fruits or vegetables	☐	☐	☐	☐	☐	☐	☐

35. How often do you usually have **breakfast** (more than a glass of milk or fruit juice)? Mark one box for <u>weekdays</u> and one box for <u>weekend</u>.

Weekdays

- ☐ I never have breakfast on weekdays
- ☐ One day
- ☐ Two days
- ☐ Three days
- ☐ Four days
- ☐ Five days

Weekend

- ☐ I never have breakfast on the weekend
- ☐ I usually have breakfast on only one day of the weekend (Saturday OR Sunday)
- ☐ I usually have breakfast on both weekend days (Saturday AND Sunday)

36. Does your school serve school lunches?

☐ Yes ☐ No

37. In the <u>last week</u> you were in school, about **how many times a week** did you eat a school lunch?

☐ 0 days ☐ 1 day ☐ 2 days ☐ 3 days ☐ 4 days ☐ 5 days

38. During the <u>past week</u>**,** how many meals (breakfast, lunch or dinner) did you get that were **prepared away from home** in places such as restaurants, fast food places, food stands, grocery stores or vending machines? (please do not include meals provided as part of school breakfast or school lunch)

ISCOLE QUALITY CONTROL (QC) and DATA ENTRY:

QC Staff Initials: ____________________________ Date: ______ / ________ / _______

Data Entry Staff Initials: ______________________ Date: ______ /________ / _______

Participant ID (attach label here)	Technician Initials ☐☐☐ Date ☐☐ / ☐☐☐ / ☐☐☐☐ (Day, Month, Year)

☐☐ meals

How well do these statements describe you? (Put a mark in the box that best describes how often this happens).

	Never or Almost Never	Sometimes	Usually or Always
39. When I am worried I eat more	☐	☐	☐
40. I eat when I am mad	☐	☐	☐
41. When I do something well I give myself a food treat	☐	☐	☐
42. When I am sad I eat more	☐	☐	☐
43. When I am happy I eat more	☐	☐	☐
44. When I am bored I eat more	☐	☐	☐
**45.** I eat between meals even when I am not hungry	☐	☐	☐

Thinking about the last week….. (Put a mark in the box that best describes how you felt)

	Not at all	Slightly	Moderately	Very	Extremely
46. Have you felt fit and well?	☐	☐	☐	☐	☐
47. Have you felt full of energy?	☐	☐	☐	☐	☐
48. Have you felt sad?	☐	☐	☐	☐	☐
49. Have you felt lonely?	☐	☐	☐	☐	☐
50. Have you had enough time for yourself?	☐	☐	☐	☐	☐
51. Have you been able to do the things that you want to do in your free time?	☐	☐	☐	☐	☐
52. Have your parent(s) treated you fairly?	☐	☐	☐	☐	☐
53. Have you had fun with your friends?	☐	☐	☐	☐	☐
54. Have you got on well at school?	☐	☐	☐	☐	☐
55. Have you been able to pay attention?	☐	☐	☐	☐	☐

56. In general, how would you say your health is?

☐ excellent ☐ very good ☐ good ☐ fair ☐ poor

ISCOLE QUALITY CONTROL (QC) and DATA ENTRY:

QC Staff Initials: ______________________________ Date: ______ / ________ / _______

Data Entry Staff Initials: ________________________ Date: ______ /________ / _______

Participant ID
(attach label here)

Technician Initials | | | |

Date [ ][ ] / [ ][ ][ ] / [ ][ ][ ][ ]
Day Month Year

Thank you

ISCOLE QUALITY CONTROL (QC) and DATA ENTRY:

QC Staff Initials: ______________________________ Date: ______ / ________ / _______

Data Entry Staff Initials: ________________________ Date: ______ /________ / _______
