# Supplementary material for: Active streets for children: The case of the Bogotá Ciclovía
Source: PLoS One. 2019 May 15;14(5):e0207791. doi: 10.1371/journal.pone.0207791 (PMC6519789; doi:10.1371/journal.pone.0207791)
Supplement: S3 File — (PDF) [file pone.0207791.s003.pdf]

Participant ID
(attach label here)

Technician Initials ☐☐☐

Date ☐☐ / ☐☐☐ / ☐☐☐☐
Day Month Year

APPENDIX H: ISCOLE Anthropometric Data Collection Form

1. Standing Height

1. ⌴ ⌴ ⌴ . ⌴ cm
2. ⌴ ⌴ ⌴ . ⌴ cm
3. ⌴ ⌴ ⌴ . ⌴ cm

2. Sitting Height

Table/Box Height:

1. ⌴ ⌴ ⌴ . ⌴ cm

Total Sitting Height:

1. ⌴ ⌴ ⌴ . ⌴ cm
2. ⌴ ⌴ ⌴ . ⌴ cm
3. ⌴ ⌴ ⌴ . ⌴ cm

☐ **Check if PT could not remove head attire for height measurements**

3. Mid-Upper-Arm Circumference

1. ⌴ ⌴ ⌴ . ⌴ cm
2. ⌴ ⌴ ⌴ . ⌴ cm
3. ⌴ ⌴ ⌴ . ⌴ cm

4. Waist Circumference

1. ⌴ ⌴ ⌴ . ⌴ cm
2. ⌴ ⌴ ⌴ . ⌴ cm
3. ⌴ ⌴ ⌴ . ⌴ cm

5. Weight

1. ⌴ ⌴ ⌴ . ⌴ kg
2. ⌴ ⌴ ⌴ . ⌴ kg
3. ⌴ ⌴ ⌴ . ⌴ kg

6. Body Fat

1. ⌴ ⌴ . ⌴ %
2. ⌴ ⌴ . ⌴ %
3. ⌴ ⌴ . ⌴ %

7. Impedance

1. ⌴ ⌴ ⌴ . ⌴ Ω
2. ⌴ ⌴ ⌴ . ⌴ Ω
3. ⌴ ⌴ ⌴ . ⌴ Ω

☐ **Check if PT is wearing socks/hosiery for weight and body fat measurements**

ISCOLE QUALITY CONTROL (QC) and DATA ENTRY:

QC Staff Initials: ______________________________ Date: ______ / ________ / _______

Data Entry Staff Initials: ________________________ Date: ______ /________ / _______
