# Supplementary material for: Active streets for children: The case of the Bogotá Ciclovía
Source: PLoS One. 2019 May 15;14(5):e0207791. doi: 10.1371/journal.pone.0207791 (PMC6519789; doi:10.1371/journal.pone.0207791)
Supplement: S4 File — (DOCX) [file pone.0207791.s004.docx]

**APÉNDICE E: Cuestionario de ISCOLE sobre salud familiar y datos demográficos**

**A. INFORMACIÓN GENERAL**

Nombre del niño/niña:

Primer apellido Segundo Apellido Primer nombre Segundo nombre

Nombre del colegio al que asiste el niño/niña:

Nombre del padre o tutor legal del niño:

Primer apellido Segundo Apellido Primer nombre Segundo nombre

Nombre de la madre o tutora legal del niño:

Primer apellido Segundo Apellido Primer nombre Segundo nombre

Dirección de residencia:

Calle Apt. # Ciudad Departamento

Intersección entre calle y carrera más cercana a la casa:

Número de teléfono ó celular (preferiblemente celular): __ __ __ __ _­_ __ __ __ __ __

Código del área: 57

E-mail:

¿Hace cuánto vive en la dirección actual? años y meses

**B. DATOS DEMOGRÁFICOS DEL NIÑO/NIÑA**

Fecha de nacimiento _____/_____/____ Edad _____años Género: ⬜ Masculino ⬜ Femenino

dd/mmm/aaaa

**Ejemplo: 02/JUN/2001**

Etnia:

**Instrucción para encuestadora:** lea todas las opciones de respuesta.

- Indígena
- Gitano(Rom)
- Raizal del archipiélago
- Palenquero de San Basilio
- Negro(a) Mulato(a)/afrocolombiano/afrodescendiente
- Ninguna de las anteriores
- Otra

¿Es usted de origen hispano? Sí No No sé

¿En qué país nació el niño/niña?

¿Cuántos(as) hermanos(as) biológicos tiene el niño/niña?

¿Cuáles son sus edades? _____años _____años _____años _____años _____años _____años _____años _____años _____años _____años

**C. HISTORIA DE LA SALUD DEL NIÑO/NIÑA**

**Instrucción para encuestadora:**

**Si la madre no sabe el peso del niño al nacer pedirle el carnet de vacunación para verificar. Consignar el peso y la talla tal y como los refiere el padre de familia.**

1. Peso al nacer: ________kilogramos y ______ gramos ó _______libras y _____onzas

Estatura al nacer: _______centímetros ó _______pulgadas

**2.** Duración del embarazo: _______semanas ó ____________meses

**3.** ¿Desarrolló la madre diabetes gestacional cuando estaba embarazada con **ESTE** niño? ⬜Sí ⬜ No

**4.**¿Se le dió leche materna? Sí No Si No, por favor pase a la pregunta 5.

**Instrucción para encuestadora:**

**Si la madre no sabe qué es fórmula láctea decirle que es leche de tarro.**

**(Si se dejó de dar leche materna o se empezó a dar leche de tarro ó fórmula láctea desde los primeros días de vida escribir <1 mes)**

Edad a la que se le dejó de dar leche materna **COMPLETAMENTE**: _____meses

Edad a la que se le dió leche de tarro ó fórmula láctea infantil por **PRIMERA VEZ**:____ meses

**5.** Edad a la que dejó de tomar leche de tarro ó fórmula láctea infantil **COMPLETAMENTE**: _____meses

**C. SALUD Y DATOS DEMOGRÁFICOS DE LA FAMILIA**

**6.** ¿Cuál es el estado civil de los padres del niño/niña?

- Casados
- Divorciados o separados
- Nunca se casaron
- Padre o madre viudo

**7.** ¿Cuántas personas viven en su hogar? (en esta dirección) ­__________

7a. ¿Quien vive con el niño(a) **en esta dirección**? (marque todas las que apliquen)

| - Madre biológica - Padre biológico - Madre adoptiva - Padre adoptivo - Madrastra - Padrastro | - Hermano (s) hermana (s) - Abuelo (s) - Otro (s) pariente (s) - Amigos (s) - Tutor (es) legal (es) (quien tiene la custodia del niño si no es ninguna de las opciones anteriores) - OTRO |
| --- | --- |

**8.** ¿Cuáles son los ingresos anuales **COMBINADOS** de su hogar (**Lea:** antes de impuestos)?

**Instrucción para encuestadora:** lea todas las opciones de respuesta. Si la persona no sabe cuánto son los ingresos anuales combinados del hogar, indague por los ingresos mensuales y haga el cálculo. (Recuerde que el valor del salario mínimo para el 2012 es: $ 566,700 pesos)

- $0 - $1.200.000
- $1.200.001 - $4.800.000
- $4.800.001- $8.400.000
- $8.400.001- $12.000.000
- $12.000.001- $18.000.000
- $18.000.001- $24.000.000
- $24.000.001- $36.000.000
- $36.000.001 en adelante
- $36.000.001 - $54.000.000
- $54.000.001 en adelante

**9.** ¿Cuántos vehículos motorizados (carro, camión, moto, ciclomotor) hay en buenas condiciones y disponibles para usar en su casa?

- 0
- 1
- 2
- 3
- 4
- 5 o más

**10.** ¿Cuántos televisores tienen en su hogar?

- 0
- 1
- 2
- 3
- 4
- 5 o más

**11.** Describa cuál es el tipo de servicio de televisión instalado en el televisor **principal** de su casa.

**Instrucción para encuestadora:** lea todas las opciones de respuesta.

- No tiene televisor
- Sólo antena
- Servicio básico de TV por cable
- Servicio de TV por cable + paquete Premium de canal(es)
- Antena parabólica
- Otra
- No sabe

**12.** ¿Describa cuál es el tipo de servicio de Internet que tiene?

**Instrucción para encuestadora:** lea todas las opciones de respuesta.

- No tiene acceso a Internet
- Módem por teléfono (internet conectado por medio de línea telefónica)
- Modelo de DSL (NOTA: no existe en Colombia)
- Módem por cable (internet conectado por medio de cable de Televisión)
- Otra
- No sabe

**13.** ¿Cuál es el nivel educativo más alto completado por la **MADRE**?

**Instrucción para encuestadora:** lea todas las opciones de respuesta.

- Inferior a la secundaria/bachillerato
- Algunos estudios de secundaria/bachillerato pero no completos
- Diploma de bachiller/grado en educación secundaria
- Licenciatura/título técnico ó 1-3 años de universidad
- Diploma de pregrado
- Posgrado

**14. ¿**Cuántas horas semanales trabaja la **MADRE** por fuera de la casa?

**Instrucción para encuestadora:** lea todas las opciones de respuesta.

- Ninguna
- Menos de 24 horas semanales
- Tiempo parcial (24-48 horas semanales)
- Tiempo completo (48+ horas semanales)

**15.** ¿Cuál es el nivel educativo más alto completado por el **PADRE**?

**Instrucción para encuestadora:** lea todas las opciones de respuesta.

- Inferior a la secundaria/bachillerato
- Algunos estudios de secundaria/bachillerato pero no completos
- Diploma de bachiller/grado en educación secundaria
- Licenciatura/título técnico ó 1-3 años de universidad
- Diploma de pregrado
- Posgrado

**16. ¿**Cuántas horas semanales trabaja el **PADRE** por fuera de la casa?

**Instrucción para encuestadora:** lea todas las opciones de respuesta.

- Ninguna
- Menos de 24 horas semanales
- Tiempo parcial (24-48 horas semanales)
- Tiempo completo (48+ horas semanales)

**17.** ¿El (la) niño(a) es adoptado(a)?

🞎 Sí (**Instrucción para encuestadora:** Pase al apéndice F)

🞎 No

**18.** Por favor responda las siguientes preguntas con respecto a la **MADRE BIOLÓGICA** del niño/niña:

**Instrucción para encuestadora:** Cuando no se pueda entrevistar a la madre biológica solicitar el teléfono para verificar los datos

Estatura actual: ______metros y ______centímetros ó _____ pies y _____ pulgadas

Peso actual: _______kilogramos ó _____libras

Edad actual: _____ años

Edad a la que tuvo o nació el niño/niña: _____ años

- La información de la madre biológica no puede ser estimada o no se sabe

**19.** Por favor responda las siguientes preguntas con respecto al **PADRE BIOLÓGICO** del niño/niña:

**Instrucción para encuestadora:** Cuando no se pueda entrevistar al padre biológico solicitar el teléfono para verificar los datos

Estatura actual: ______metros y ______centímetros ó _____ pies y _____ pulgadas

Peso actual: _______kilogramos ó _____libras

Edad actual: _____ años

- La información del padre biológico no puede ser estimada o no se sabe

**APÉNDICE F: Cuestionario de información para contacto y seguimiento**

**Instrucción para encuestadora. Lea:**

Puede ser que los investigadores de este estudio deseen ponerse en contacto con usted nuevamente para obtener información adicional relacionada con su salud y la de su hijo(a). Por favor díganos el nombre, la dirección, el número de teléfono y la dirección de correo electrónico de dos parientes o amigos que sabrían adónde llamarlo en caso de que tengamos dificultad en contactarlos. (**lea:**Por favor denos los nombres de 2 personas que no viven actualmente en su casa.)

**Persona 1**

| Nombre  Primer apellido Segundo Apellido Primer nombre Segundo nombre  Dirección:  Calle Apartamento # Ciudad Departamento Código Postal País  Número de teléfono: ( ) Celular: E-Mail:  Código del área: 57 |
| --- |

**¿Qué parentesco tiene con la persona de contacto?**

⬜ Cónyuge/ex-cónyuge que NO vive en su casa ⬜ Tutor legal (quien tiene la custodia del niño si no es ninguna de las opciones anteriores)

⬜Pareja no casada que NO vive en su casa ⬜Amigo(a)

⬜ Padre o madre ⬜ Compañero(a) de trabajo

⬜ Hermano o hermana ⬜ Vecino(a)

⬜ Abuelo(a) ⬜ OTRO

⬜ Otro pariente

**Persona 2**

| Nombre  Primer apellido Segundo Apellido Primer nombre Segundo nombre  Dirección:  Calle Apartamento # Ciudad Departamento Código Postal País  Número de teléfono: ( ) Celular: E-Mail:  Código del área: 57 |
| --- |

**¿Qué parentesco tiene con la persona de contacto?**

⬜ Cónyuge/ex-cónyuge que NO vive en su casa ⬜ Tutor legal (quien tiene la custodia del niño si no es ninguna de las opciones anteriores)

⬜ Pareja no casada que NO vive en su casa ⬜ Amigo(a)

⬜ Padre o madre ⬜ Compañero(a) de trabajo

⬜ Hermano o hermana ⬜ Vecino(a)

⬜ Abuelo(a) ⬜ OTRO

⬜ Otro pariente

**APÉNDICE N**

**CUESTIONARIO DE ISCOLE SOBRE EL BARRIO Y EL ENTORNO FAMILIAR**

**Instrucción para encuestadora. Lea:**

Donde se menciona al “niño”, por favor responda únicamente acerca del niño que está participando en este estudio. Sea lo más preciso que pueda. Ninguna respuesta es correcta o incorrecta. Toda la información es estrictamente confidencial.

**A. COHESIÓN DEL BARRIO**

| **¿Está de acuerdo o en desacuerdo con las siguientes afirmaciones?** | Totalmente en desacuerdo | Un poco en desacuerdo | Neutro | Un poco de acuerdo | Totalmente de acuerdo |
| --- | --- | --- | --- | --- | --- |
| 1. La gente de mi barrio está dispuesta a ayudar a sus vecinos. | ⭘ | ⭘ | ⭘ | ⭘ | ⭘ |
| 2. Este es un barrio muy unido. | ⭘ | ⭘ | ⭘ | ⭘ | ⭘ |
| 3. La gente de mi barrio es confiable. | ⭘ | ⭘ | ⭘ | ⭘ | ⭘ |
| 4. La gente de mi barrio por lo general no se la lleva bien. | ⭘ | ⭘ | ⭘ | ⭘ | ⭘ |
| 5. La gente de mi barrio no comparte los mismos valores, actitudes o creencias. | ⭘ | ⭘ | ⭘ | ⭘ | ⭘ |

**B. VECINOS Y AMIGOS**

1. Piense en el barrio o el sector en donde vive. En general, ¿qué tanto cree conocer a sus vecinos?

**Instrucción para encuestadora:** lea todas las opciones de respuesta.

| En lo absoluto | Sólo un poco | Moderadamente bien | Muy bien |
| --- | --- | --- | --- |
| ⭘ | ⭘ | ⭘ | ⭘ |

2. Aproximadamente, ¿qué tan frecuentemente habla con o visita a sus vecinos inmediatos (la gente que vive en las 10-20 hogares/casas más cercanas a la suya)?

**Instrucción para encuestadora:** lea todas las opciones de respuesta.

| Nunca | Una vez al año o menos | Varias veces al año | Una vez al mes | Varias veces al mes | Varias veces a la semana | Casi todos los días |
| --- | --- | --- | --- | --- | --- | --- |
| ⭘ | ⭘ | ⭘ | ⭘ | ⭘ | ⭘ | ⭘ |

**C. RESPUESTAS ACERCA DEL BARRIO**

**Instrucción para encuestadora:** Con cada opción de pregunta lea todas las opciones de respuesta.

| **Para las siguientes afirmaciones, por favor marque qué tan probable sería que un vecino reaccionara a, o emprendiera alguna acción ante las siguientes situaciones:** | Bastante improbable | Improbable | Ni probable o improbable | Probable | Muy probable |
| --- | --- | --- | --- | --- | --- |
| 1. Si un grupo de niños del barrio faltara a la escuela y estuviera pasando el tiempo en la esquina del barrio, ¿qué tan probable sería que sus vecinos hicieran algo al respecto? | ⭘ | ⭘ | ⭘ | ⭘ | ⭘ |
| 2. Si un grupo de niños estuviera pintando grafitis en un local o un edificio del barrio, ¿qué tan probable sería que sus vecinos hicieran algo al respecto? | ⭘ | ⭘ | ⭘ | ⭘ | ⭘ |
| 3. Si un niño estuviera irrespetando a un adulto, ¿qué tan probable sería que sus vecinos regañaran al niño? | ⭘ | ⭘ | ⭘ | ⭘ | ⭘ |
| 4. Si hubiera una pelea frente a su casa y estuvieran golpeando o amenazando a alguien, ¿qué tan probable sería que sus vecinos intervinieran para detener la pelea? | ⭘ | ⭘ | ⭘ | ⭘ | ⭘ |
| 5. Suponga que debido a falta de presupuesto fuesen a cerrar la estación de policía más próxima a su casa. ¿Qué tan probable sería que los residentes del barrio se unieran para tratar de mantener la estación de policía funcionando? | ⭘ | ⭘ | ⭘ | ⭘ | ⭘ |

**D. ALIMENTOS EN CASA**

**Instrucción para encuestadora:** Con cada opción de pregunta lea todas las opciones de respuesta.

| **¿Con qué frecuencia están disponibles los siguientes alimentos/bebidas en su casa?** | Nunca | Rara vez | A veces | Casi siempre | Siempre |
| --- | --- | --- | --- | --- | --- |
| 1. Chocolates | ⭘ | ⭘ | ⭘ | ⭘ | ⭘ |
| 2. Otros dulces | ⭘ | ⭘ | ⭘ | ⭘ | ⭘ |
| 3. Fruta (por ejemplo: manzanas, naranjas) | ⭘ | ⭘ | ⭘ | ⭘ | ⭘ |
| 4. Ponqués, brownies, muffins o galletas | ⭘ | ⭘ | ⭘ | ⭘ | ⭘ |
| 5. Papas fritas normales de paquete o galletas de sal | ⭘ | ⭘ | ⭘ | ⭘ | ⭘ |
| 6. Papas horneadas de paquete, galletas de sal bajas en grasa, rosquillas de sal o pretzels | ⭘ | ⭘ | ⭘ | ⭘ | ⭘ |
| 7. Verduras crudas (por ejemplo: zanahorias) | ⭘ | ⭘ | ⭘ | ⭘ | ⭘ |
| 8. Jugo de pura fruta (100% fruta) | ⭘ | ⭘ | ⭘ | ⭘ | ⭘ |
| 9. Bebidas de fruta (por ejemplo: Tampico, jugos Hit, Jugos del Valle, Tutti Frutti) | ⭘ | ⭘ | ⭘ | ⭘ | ⭘ |
| 10. Gaseosas regulares con azúcar | ⭘ | ⭘ | ⭘ | ⭘ | ⭘ |
| 11. Gaseosas dietéticas o sin azúcar | ⭘ | ⭘ | ⭘ | ⭘ | ⭘ |
| 12. Bebidas deportivas (por ejemplo: Gatorade) | ⭘ | ⭘ | ⭘ | ⭘ | ⭘ |
| 13. Rollos de fruta u otras frutas deshidratadas | ⭘ | ⭘ | ⭘ | ⭘ | ⭘ |
| 14. Leche entera o al 2% | ⭘ | ⭘ | ⭘ | ⭘ | ⭘ |
| **¿Con qué frecuencia están disponibles los siguientes alimentos/bebidas en su casa?** | Nunca | Rara vez | A veces | Casi siempre | Siempre |
| 15. Leche al 1% o descremada | ⭘ | ⭘ | ⭘ | ⭘ | ⭘ |
| 16. Cereal endulzado para el desayuno | ⭘ | ⭘ | ⭘ | ⭘ | ⭘ |
| 17. Cereal sin azúcar para el desayuno | ⭘ | ⭘ | ⭘ | ⭘ | ⭘ |

**E. LUGARES DONDE COMPRA SUS ALIMENTOS**

**Instrucción para encuestadora:** Con cada opción de pregunta lea todas las opciones de respuesta.

| **Cuando usted, o quien normalmente compra la comida en su casa, van a comprar la comida, ¿con qué frecuencia van a cada uno de los siguientes tipos de tiendas?** | Nunca | Rara vez | A veces | Casi siempre | Siempre |
| --- | --- | --- | --- | --- | --- |
| 1. Supermercado grande o hipermercado | ⭘ | ⭘ | ⭘ | ⭘ | ⭘ |
| 2. Tiendas de comida pequeñas a medianas | ⭘ | ⭘ | ⭘ | ⭘ | ⭘ |
| 3. Tienda mediana donde venden de todo | ⭘ | ⭘ | ⭘ | ⭘ | ⭘ |
| 4. Plazas de mercado/puestos de frutas y verduras frescas | ⭘ | ⭘ | ⭘ | ⭘ | ⭘ |
| 5. Otras, detallar: __________________________ | ⭘ | ⭘ | ⭘ | ⭘ | ⭘ |

**F. ACCESO A LAS TIENDAS**

| **Por favor indique si las siguientes afirmaciones son verdaderas con respecto a la tienda donde normalmente compra sus comestibles o alimentos .** | Sí | No | No aplica |
| --- | --- | --- | --- |
| 1. Cerca a mi sitio de trabajo | ⭘ | ⭘ | ⭘ |
| 2. Cerca al colegio de mi hijo | ⭘ | ⭘ | ⭘ |
| 3. Cerca de mi casa | ⭘ | ⭘ | ⭘ |

**G. COMPRA DE ALIMENTOS**

**Instrucción para encuestadora:** Con cada opción de pregunta lea todas las opciones de respuesta.

| **Las siguientes preguntas se refieren a la tienda donde normalmente compra su comida.** | Totalmente en desacuerdo | Un poco en desacuerdo | Neutro | Un poco de acuerdo | Totalmente de acuerdo |
| --- | --- | --- | --- | --- | --- |
| 1. Las comidas bajas en grasa son demasiado costosas. | ⭘ | ⭘ | ⭘ | ⭘ | ⭘ |
| 2. Hay gran variedad de frutas y verduras frescas disponibles. | ⭘ | ⭘ | ⭘ | ⭘ | ⭘ |
| 3. Hay gran variedad de productos bajos en grasa disponibles. | ⭘ | ⭘ | ⭘ | ⭘ | ⭘ |
| 4. Mala condición de frutas y verduras. | ⭘ | ⭘ | ⭘ | ⭘ | ⭘ |
| 5. Las frutas y verduras son demasiado costosas. | ⭘ | ⭘ | ⭘ | ⭘ | ⭘ |

**H. APARATOS ELECTRÓNICOS DE SU HIJO**

| **Por favor indique si su hijo tiene los siguientes aparatos en su habitación.** | Sí | No |
| --- | --- | --- |
| 1. Televisor | ⭘ | ⭘ |
| 2. Computador | ⭘ | ⭘ |
| 3. Sistema de juegos de video (no portátiles; Playstation, Xbox, etc.) | ⭘ | ⭘ |
|  |  |  |
| **¿Posee su hijo los siguientes artículos para su uso personal?** |  |  |
| 4. Teléfono celular o radio transmisores | ⭘ | ⭘ |
| 5. Reproductores de juegos de video portátiles (Game Boy, Sony PSP, etc.) | ⭘ | ⭘ |
| 6. Reproductores de Música (Ipod, grabadora, radio, etc.) | ⭘ | ⭘ |

**Para las dos preguntas siguientes, por favor piense en las actividades de su hijo durante el *último año*.**

1. **ARTÍCULOS DE DIVERSIÓN**

**Instrucción para encuestadora:** Con cada opción de pregunta lea todas las opciones de respuesta.

| **¿Con qué frecuencia durante el último año usó su hijo los siguientes artículos en su casa o cerca de ésta (o en un área común de su apartamento o conjunto)?** | No disponible (no tiene) | Disponible pero nunca lo usa | Una vez al mes o menos | Una vez cada dos semanas | Una vez a la semana o más |
| --- | --- | --- | --- | --- | --- |
| 1. Bicicleta | ⭘ | ⭘ | ⭘ | ⭘ | ⭘ |
| 2. Canasta o cesta de baloncesto | ⭘ | ⭘ | ⭘ | ⭘ | ⭘ |
| 3. Lazo para saltar | ⭘ | ⭘ | ⭘ | ⭘ | ⭘ |
| 4. Videojuegos activos (por ejemplo: tapete para bailar, Wii, etc.) | ⭘ | ⭘ | ⭘ | ⭘ | ⭘ |
| 5. Artículos deportivos (como pelotas, raquetas, bates, palos) | ⭘ | ⭘ | ⭘ | ⭘ | ⭘ |
| 6. Piscina | ⭘ | ⭘ | ⭘ | ⭘ | ⭘ |
| 7. Patines, patineta o monopatín | ⭘ | ⭘ | ⭘ | ⭘ | ⭘ |
| 8. Equipos de juego fijos (por ejemplo: columpios, casa de muñecas, pasamanos) | ⭘ | ⭘ | ⭘ | ⭘ | ⭘ |

**J. LUGARES DONDE SU HIJO HACE ACTIVIDAD FÌSICA**

**Actividad física es cualquier actividad que aumente los latidos de su corazón y la respiración.**

**Instrucción para encuestadora:** Con cada opción de pregunta lea todas las opciones de respuesta.

| **¿Con qué frecuencia durante el último año su hijo fue activo físicamente (incluyendo juegos activos) en los siguientes lugares?** | Nunca | Una vez al mes o menos | Una vez cada dos semanas | Una vez a la semana | 2 ó 3 veces por semana | 4 veces por semana o más |
| --- | --- | --- | --- | --- | --- | --- |
| 1. Dentro de la casa | ⭘ | ⭘ | ⭘ | ⭘ | ⭘ | ⭘ |
| 2. En su jardín o zona común o en el camino de entrada a la casa | ⭘ | ⭘ | ⭘ | ⭘ | ⭘ | ⭘ |
| 3. En la casa, el jardín o en el camino de entrada a la casa de un vecino | ⭘ | ⭘ | ⭘ | ⭘ | ⭘ | ⭘ |
| 4. En una calle, andén o lote baldío del barrio | ⭘ | ⭘ | ⭘ | ⭘ | ⭘ | ⭘ |
| 5. Centro de recreación bajo techo o gimnasio (público o privado; por ejemplo: Club de Niños y Niñas) | ⭘ | ⭘ | ⭘ | ⭘ | ⭘ | ⭘ |
| 6. Playa, laguna, río o quebrada | ⭘ | ⭘ | ⭘ | ⭘ | ⭘ | ⭘ |
| 7. Ciclorutas, caminos para caminar y trotar | ⭘ | ⭘ | ⭘ | ⭘ | ⭘ | ⭘ |
| 8. Cancha de baloncesto | ⭘ | ⭘ | ⭘ | ⭘ | ⭘ | ⭘ |
| 9. Otras canchas/campos de juego (como fútbol, softbol, tenis) | ⭘ | ⭘ | ⭘ | ⭘ | ⭘ | ⭘ |
| 10. Parque público pequeño o parque infantil | ⭘ | ⭘ | ⭘ | ⭘ | ⭘ | ⭘ |
| 11. Parque público grande | ⭘ | ⭘ | ⭘ | ⭘ | ⭘ | ⭘ |
| 12. Espacio público al aire libre distinto de un parque | ⭘ | ⭘ | ⭘ | ⭘ | ⭘ | ⭘ |
| 13. Predios de la escuela (por fuera de la jornada escolar) | ⭘ | ⭘ | ⭘ | ⭘ | ⭘ | ⭘ |

**K. MOVILIZACIÓN DENTRO DE SU BARRIO**

**Instrucción para encuestadora:** Con cada opción de pregunta lea todas las opciones de respuesta.

| **Por favor seleccione la respuesta que más se ajusta a usted y a su barrio. Una distancia caminable significa que le toma entre 10 a 15 minutos ir a pie desde su casa.** | | | | |
| --- | --- | --- | --- | --- |
|  | Totalmente en desacuerdo | Un poco en desacuerdo | Un poco de acuerdo | Totalmente de acuerdo |
| 1. A una distancia caminable de mi casa hay tiendas, almacenes, supermercados y lugares para comprar lo que necesito. | ⭘ | ⭘ | ⭘ | ⭘ |
| 2. A una distancia caminable de mi casa hay un paradero de bus o estación de TransMilenio. | ⭘ | ⭘ | ⭘ | ⭘ |
| 3. Casi todas las calles tienen andén. | ⭘ | ⭘ | ⭘ | ⭘ |
| 4. No hay muchas calles cerradas. | ⭘ | ⭘ | ⭘ | ⭘ |
| 5. Hay varias rutas diferentes para desplazarse de un lugar a otro. | ⭘ | ⭘ | ⭘ | ⭘ |
| 6. La tasa de criminalidad es alta. | ⭘ | ⭘ | ⭘ | ⭘ |
| 7. Los automóviles normalmente andan despacio en la mayoría de las calles (a 48 km/hr o menos). | ⭘ | ⭘ | ⭘ | ⭘ |
| 8. Casi todos los conductores exceden el límite de velocidad. | ⭘ | ⭘ | ⭘ | ⭘ |
| 9. Puedo ver varias cosas interesantes mientras camino por mi barrio. | ⭘ | ⭘ | ⭘ | ⭘ |
| 10. El tráfico dificulta o hace que mi hijo no pueda caminar tranquilamente. | ⭘ | ⭘ | ⭘ | ⭘ |
| 11. Las calles están bien iluminadas por la noche. | ⭘ | ⭘ | ⭘ | ⭘ |
| 12. Las calles más transitadas cuentan con cruces peatonales y señalización. | ⭘ | ⭘ | ⭘ | ⭘ |
| 13. A una distancia caminable de mi casa existen muchos lugares para ir. | ⭘ | ⭘ | ⭘ | ⭘ |
| 14. Temo que algún desconocido se lleve o lastime a mi hijo en las calles del barrio. | ⭘ | ⭘ | ⭘ | ⭘ |
| 15. Temo que algún desconocido se lleve o lastime a mi hijo en mi jardín, la entrada de mi casa o una zona común. | ⭘ | ⭘ | ⭘ | ⭘ |
| 16. Temo que algún desconocido se lleve o lastime a mi hijo en un parque local. | ⭘ | ⭘ | ⭘ | ⭘ |
| 17. Temo que alguna persona malintencionada (adulto o niño) se lleve o lastime a mi hijo dentro del barrio. | ⭘ | ⭘ | ⭘ | ⭘ |

**L. DISTANCIA A CIERTOS LUGARES**

**Instrucción para encuestadora:** Con cada opción de pregunta lea todas las opciones de respuesta.

| **¿ Aproximadamente cuánto tiempo le tomaría a usted caminar desde su casa hasta los lugares más cercanos listados a continuación? Por favor seleccione el tiempo que le tomaría ir a pie a cada lugar, independientemente de si frecuentan ese lugar usted y/o su hijo.** | | | | | | |
| --- | --- | --- | --- | --- | --- | --- |
|  | 1-5 min | 6-10 min | 11-20 min | 21-30 min | 31+ min | No sabe |
| 1. Tienda de abarrotes/tienda de la esquina, mini mercado, bodega | ⭘ | ⭘ | ⭘ | ⭘ | ⭘ | ⭘ |
| 2. Supermercado | ⭘ | ⭘ | ⭘ | ⭘ | ⭘ | ⭘ |
| 3. Restaurante de comida rápida | ⭘ | ⭘ | ⭘ | ⭘ | ⭘ | ⭘ |
| 4. Restaurante que no sea de comida rápida | ⭘ | ⭘ | ⭘ | ⭘ | ⭘ | ⭘ |
| 5. Centro de recreación bajo techo o gimnasio (público o privado; por ejemplo: Club de Niños &Niñas) | ⭘ | ⭘ | ⭘ | ⭘ | ⭘ | ⭘ |
| 6. Playa, laguna, río o quebrada | ⭘ | ⭘ | ⭘ | ⭘ | ⭘ | ⭘ |
| 7. Ciclorutas, senderos para caminar y trotar | ⭘ | ⭘ | ⭘ | ⭘ | ⭘ | ⭘ |
| 8. Cancha de baloncesto | ⭘ | ⭘ | ⭘ | ⭘ | ⭘ | ⭘ |
| 9. Otras canchas/campos de juego (como fútbol, softbol, tenis, parque para caminar) | ⭘ | ⭘ | ⭘ | ⭘ | ⭘ | ⭘ |
| 10. Parque público pequeño | ⭘ | ⭘ | ⭘ | ⭘ | ⭘ | ⭘ |
| 11. Parque público grande | ⭘ | ⭘ | ⭘ | ⭘ | ⭘ | ⭘ |
| 12. Espacio público abierto diferente a un parque | ⭘ | ⭘ | ⭘ | ⭘ | ⭘ | ⭘ |
| 13. Escuela con instalaciones recreativas de uso público | ⭘ | ⭘ | ⭘ | ⭘ | ⭘ | ⭘ |

**M. FAMILIA**

**Instrucción para encuestadora:** Con cada opción de pregunta lea todas las opciones de respuesta.

| **Durante una semana típica, ¿con qué frecuencia usted u otro adulto en el hogar?:** | | | | | |
| --- | --- | --- | --- | --- | --- |
|  | Nunca | 1-2 días | 3-4 días | 5-6 días | Todos los días |
| 1. Ve a su hijo/hija mientras hace actividad física o practica algún deporte | ⭘ | ⭘ | ⭘ | ⭘ | ⭘ |
| 2. Estimula a su hijo/hija para que practique un deporte o haga actividad física | ⭘ | ⭘ | ⭘ | ⭘ | ⭘ |
| 3. Lleva o transporta a su hijo/hija hasta un lugar donde pueda hacer actividad física o practicar un deporte | ⭘ | ⭘ | ⭘ | ⭘ | ⭘ |
| 4. Hace actividad física o practica un deporte con su hijo/hija | ⭘ | ⭘ | ⭘ | ⭘ | ⭘ |

**APÉNDICE Q**

**CUESTIONARIO SOBRE CICLOVÍAS**

**Instrucción para encuestadora. Lea:**

Donde se menciona al “niño”, por favor responda únicamente acerca del niño que está participando en este estudio. Sea lo más preciso que pueda. Ninguna respuesta es correcta o incorrecta. Toda la información es estrictamente confidencial.

1. ¿Cuál es el estrato que aparece en el recibo de la energía que llega a su casa?

| 0 (No le llega recibo) | 1 | 2 | 3 | 4 | 5 | 6 |
| --- | --- | --- | --- | --- | --- | --- |

1. ¿Con qué frecuencia USTED asiste a la Ciclovía? **(Leer, R.U.)**

| Por lo menos una vez al año | 01 |
| --- | --- |
| 1 día/mes | 02 |
| 2 días/mes | 03 |
| 3 días/mes | 04 |
| 4 días/mes | 05 |
| Siempre | 06 |
| Nunca va a la ciclovia | 07 |

1. ¿Con qué frecuencia su hijo asiste a la Ciclovía? **(Leer, R.U.)**

| Por lo menos una vez al año | 01 |
| --- | --- |
| 1 día/mes | 02 |
| 2 días/mes | 03 |
| 3 días/mes | 04 |
| 4 días/mes | 05 |
| Siempre | 06 |
| Nunca va a la ciclovia | 07 |

Si contesta nunca fin de la entrevista.

1. ¿Qué medio usa su hijo **usualmente** cuando va a la Ciclovía? **MR.**

| Bicicleta | 1 |
| --- | --- |
| Patines | 2 |
| Camina | 3 |
| Trota | 4 |
| Scooter | 5 |
| Patineta | 6 |
| Silla de ruedas | 7 |
| Otro, ¿Cuál? | |

1. ¿En promedio de horas, cuánto tiempo permanece su hijo en la Ciclovía **usualmente**?

______ HORAS

1. ¿En promedio de horas, cuánto tiempo permanece en la Ciclovía  **realizando la o las actividad que mencionó en la pregunta 4**?

______ HORAS
