# Supplementary material for: Active streets for children: The case of the Bogotá Ciclovía
Source: PLoS One. 2019 May 15;14(5):e0207791. doi: 10.1371/journal.pone.0207791 (PMC6519789; doi:10.1371/journal.pone.0207791)
Supplement: S5 File — (PDF) [file pone.0207791.s005.pdf]

ID Participante	Iniciales Encuestador ☐☐☐<br>Fecha ☐☐ / ☐☐☐ / ☐☐☐☐<br>Día Mes Año

Cuestionario administrado por: Encuestadora ☐ Auto administrado ☐

Apéndice M:
Cuestionario de ISCOLE sobre estilo de vida y nutrición

Instrucción para encuestadora: Por favor lea cada pregunta completamente. Seleccione la casilla que mejor se ajusta a la respuesta del participante y llénela.

Leer: Por favor recuerda que esta encuesta no se trata de un examen, por lo tanto no hay respuestas incorrectas. Es importante que respondas todas las preguntas y que me indiques claramente cuál es tu respuesta. Ninguna persona que te conozca va a ver tu cuestionario o a saber tus respuestas.
Para estas preguntas por favor cuéntame acerca de lo que hiciste la semana pasada.

1. En un día escolar, ¿cuántas horas viste televisión?

☐ No vi televisión en días escolares. ☐< 1 hora ☐ 1 hora ☐ 2 horas ☐ 3 horas
☐ 4 horas ☐ 5 horas o más

2. En un día escolar, ¿cuántas horas jugaste con juegos de video o de computador, o usaste el computador para cosas que no estén relacionadas con las tareas escolares?

☐ No jugué con juegos de video o de computador ni usé el computador para cosas que no estuvieran relacionadas con las tareas escolares durante los días de colegio.

☐< 1 hora ☐ 1 hora ☐ 2 horas ☐ 3 horas ☐ 4 horas ☐ 5 horas o más

3. En un día escolar, ¿cuánto tiempo estuviste al aire libre **antes** del colegio?

☐< 1 hora ☐ 1 hora ☐ 2 horas ☐ 3 horas ☐ 4 horas ☐ 5 horas o más

4. En un día escolar, ¿cuánto tiempo estuviste al aire libre **después** del colegio y antes de la hora de dormir?

☐< 1 hora ☐ 1 hora ☐ 2 horas ☐ 3 horas ☐ 4 horas ☐ 5 horas o más

5. En un día de fin de semana, ¿cuántas horas viste televisión?

☐ No vi televisión en los días de fin de semana. ☐< 1 hora ☐ 1 hora ☐ 2 horas
☐ 3 horas ☐ 4 horas ☐ 5 horas o más

CONTROL DE CALIDAD ISCOLE Y DIGITACION DE DATOS:

Iniciales del personal de Control de Calidad: ______________________Fecha: _____ / _____ / 20_____

Iniciales Personal de Digitación: ________________________________Fecha: _____ /_____ / 20_____

ID Participante	Iniciales Encuestador ☐☐☐ Fecha ☐☐ / ☐☐☐ / ☐☐☐☐ Día Mes Año

Cuestionario administrado por: Encuestadora ☐ Auto administrado ☐

6. En un <u>día de fin de semana</u>, ¿cuántas horas jugaste con juegos de video o de computador, o usaste el computador para cosas que <u>no</u> estuvieran relacionadas con las tareas escolares?

☐ No jugué con juegos de video o de computador, ni usé el computador para cosas que <u>no</u> estuvieran relacionadas con las tareas escolares durante los <u>días de fin de semana</u>.

☐< 1 hora ☐ 1 hora ☐ 2 horas ☐ 3 horas ☐ 4 horas ☐ 5 horas o más

7. En un <u>día de fin de semana</u>, ¿cuánto tiempo estuviste al aire libre?

☐< 1 hora ☐ 1 hora ☐ 2 horas ☐ 3 horas ☐ 4 horas ☐ 5 horas o más

8. Durante la <u>última semana</u> que fuiste al colegio, ¿cuántos días recibiste clases de educación física (EF)?

☐ 0 días ☐ 1 día ☐ 2 días ☐ 3 días ☐ 4 días ☐ 5 días

9. Durante la <u>última semana</u> que fuiste al colegio, <u>la mayoría</u> de este recorrido lo hiciste:

☐ caminando
☐ montando en bicicleta, patines, patineta, monopatín
☐ en bus, TransMilenio
☐ automóvil, motocicleta o ciclomotor
☐ por otro medio ______________

10. Durante la <u>última semana</u> que fuiste al colegio, **¿CUÁNTO TIEMPO** te demoraste en el recorrido de tu casa al colegio?

☐< 5 minutos ☐ 5 - 15 minutos ☐ 16 - 30 minutos ☐ 31 minutos a 1 hora ☐>1 hora

11. Durante el <u>año pasado</u> (12 meses), ¿realizaste alguna de las siguientes actividades?
Instrucción para encuestadora: Marque todas las que apliquen

☐ Deportes en equipo ☐ clases de danza o artes marciales ☐ clases de arte o música
☐Ninguna de las anteriores

12. Durante la <u>semana pasada</u> (7 días), ¿cuántos días fuiste físicamente activo por lo menos durante <u>60 minutos</u> diarios?

Instrucción para encuestadora. Lea: Piensa en todo el tiempo que estuviste haciendo actividades que incrementaran los latidos de tu corazón y que te hicieran respirar fuerte.

☐ 0 días ☐ 1 día ☐ 2 días ☐ 3 días ☐ 4 días ☐ 5 días ☐ 6 días ☐ 7 días

CONTROL DE CALIDAD ISCOLE Y DIGITACION DE DATOS:

Iniciales del personal de Control de Calidad: ______________________Fecha: _____ / _____ / 20_____

Iniciales Personal de Digitación: ________________________________Fecha: _____ /_____ / 20_____

ID Participante	Iniciales Encuestador ☐☐☐
	Fecha ☐☐ / ☐☐☐ / ☐☐☐☐ Día Mes Año

Cuestionario administrado por: Encuestadora ☐ Auto administrado ☐

Instrucción para encuestadora. Lea:
Por favor dime la opción que más se ajuste a tu respuesta, piensa que:
-1es totalmente en desacuerdo
-2 en desacuerdo
-3 ni de acuerdo ni desacuerdo
-4 de acuerdo
-5 totalmente de acuerdo
Instrucción para encuestadora: Con cada opción de pregunta lea todas las opciones de respuesta.

	**Totalmente en desacuerdo** →				**Totalmente de acuerdo**
	**1**	**2**	**3**	**4**	**5**
**13.** Puedo ser físicamente activo durante mi tiempo libre casi todos los días.	☐	☐	☐	☐	☐
**14.** Puedo pedirle a mis padres u otro adulto que hagan actividad física conmigo	☐	☐	☐	☐	☐
**15.** Puedo ser físicamente activo durante mi tiempo libre casi todos los días, incluso en lugar de ver TV o jugar con juegos de video.	☐	☐	☐	☐	☐
**16.** Puedo ser físicamente activo durante mi tiempo libre casi todos los días, incluso si hace mucho calor o frío afuera	☐	☐	☐	☐	☐
**17.** Puedo invitar a mi mejor amigo a ser físicamente activo conmigo durante mi tiempo libre casi todos los días	☐	☐	☐	☐	☐
**18.** Puedo ser físicamente activo durante mi tiempo libre casi todos los días, incluso cuando me tengo que quedar en casa.	☐	☐	☐	☐	☐
**19.** Poseo la coordinación que necesito para ser físicamente activo en mi tiempo libre casi todos los días	☐	☐	☐	☐	☐
**20.** Puedo ser físicamente activo durante mi tiempo libre casi todos los días sin importar qué tan ocupado esté mi día	☐	☐	☐	☐	☐

CONTROL DE CALIDAD ISCOLE Y DIGITACION DE DATOS:

Iniciales del personal de Control de Calidad: ______________________Fecha: _____ / _____ / 20_____

Iniciales Personal de Digitación: ________________________________Fecha: _____ /_____ / 20_____

ID Participante	Iniciales Encuestador ☐☐☐ Fecha ☐☐ / ☐☐☐ / ☐☐☐☐ Día Mes Año

Cuestionario administrado por: Encuestadora ☐ Auto administrado ☐

Instrucción para encuestadora. Lea:
Existen muchas razones por las que la gente hace actividad física. Por favor dime que tanto aplican las siguientes razones en tu caso.

Instrucción para encuestadora: Con cada opción de pregunta lea todas las opciones de respuesta.

	nunca es cierto en mi caso	algo cierto en mi caso	algunas veces cierto en mi caso	cierto en mi caso	muy cierto en mi caso
**21.** Hago ejercicio porque otras personas me dicen que debería hacerlo.	☐	☐	☐	☐	☐
**22.** Para mí es importante hacer ejercicio con regularidad	☐	☐	☐	☐	☐
**23.** No veo porqué debo preocuparme por hacer ejercicio	☐	☐	☐	☐	☐
**24.** Me siento como un perdedor cuando no hago ejercicio en mucho tiempo	☐	☐	☐	☐	☐
**25.** Encuentro que el ejercicio es una actividad agradable	☐	☐	☐	☐	☐

26. Durante los <u>días escolares</u> de la <u>semana pasada</u>, ¿usualmente a qué hora apagaste las luces para irte a dormir?
☐☐:☐☐ AM / PM (encierre en un círculo AM ó PM)

27. Durante los <u>días escolares</u> de la <u>semana pasada</u>, ¿usualmente a qué hora te levantaste en la mañana?
☐☐:☐☐ AM / PM (encierre en un círculo AM ó PM)

28. Durante los <u>días del fin de semana pasado</u>, ¿usualmente a qué hora apagaste las luces para irte a dormir?
☐☐:☐☐ AM / PM (encierre en un círculo AM ó PM)

CONTROL DE CALIDAD ISCOLE Y DIGITACION DE DATOS:

Iniciales del personal de Control de Calidad: ______________________Fecha: _____ / _____ / 20_____

Iniciales Personal de Digitación: _________________________________Fecha: _____ /_____ / 20_____

ID Participante	Iniciales Encuestador ☐☐☐<br>Fecha ☐☐ / ☐☐☐ / ☐☐☐☐<br>Día Mes Año

Cuestionario administrado por: Encuestadora ☐ Auto administrado ☐

29. Durante los días del fin de semana pasado, ¿usualmente a qué hora te levantaste en la mañana?

☐☐:☐☐AM / PM (encierre en un círculo AM ó PM)

30. Durante la semana pasada, ¿cómo calificarías la **calidad** de tu sueño en general?

Instrucción para encuestadora. **Lea:** ¿qué tan **bien** dormiste?

☐Muy buena ☐ medianamente buena ☐ medianamente mala ☐ muy mala

31. Durante la semana pasada, ¿cómo calificarías la **cantidad** de tiempo que dormiste en general?

Instrucción para encuestadora. **Lea:** ¿Qué te parece la **cantidad** de tiempo que dormiste?

☐Muy buena ☐ medianamente buena ☐ medianamente mala ☐ muy mala

32. ¿Tienes un televisor en tu habitación?

☐ Sí ☐ No

CONTROL DE CALIDAD ISCOLE Y DIGITACION DE DATOS:

Iniciales del personal de Control de Calidad: ______________________Fecha: _____ / _____ / 20_____

Iniciales Personal de Digitación: ________________________________Fecha: _____ /_____ / 20_____

ID Participante	Iniciales Encuestador ☐☐☐
	Fecha ☐☐ / ☐☐☐ / ☐☐☐☐
	Día Mes Año

Cuestionario administrado por: Encuestadora ☐ Auto administrado ☐

33. ¿Usualmente cuántas veces a la semana comes …?

Instrucción para encuestadora:
Con cada opción de pregunta lea todas las opciones de respuesta.
Marque una sola casilla por renglón.

	Nunca	Menos de una vez a la semana	Una vez a la semana	2-4 días a la semana	5-6 días a la semana	Una vez al día, todos los días	Todos los días, más de una vez
Frutas	☐	☐	☐	☐	☐	☐	☐
Verduras	☐	☐	☐	☐	☐	☐	☐
Dulces (dulces/chocolate)	☐	☐	☐	☐	☐	☐	☐
Gaseosa regular o bebidas azucaradas	☐	☐	☐	☐	☐	☐	☐
Ponqué, bizcochos o donas	☐	☐	☐	☐	☐	☐	☐
Gaseosa de dieta o bebidas azucaradas de dieta	☐	☐	☐	☐	☐	☐	☐
Papas fritas de paquete	☐	☐	☐	☐	☐	☐	☐
Papas a la francesa	☐	☐	☐	☐	☐	☐	☐
Verduras color verde oscuro (brócoli, espinaca, acelga, etc.)	☐	☐	☐	☐	☐	☐	☐
Verduras color naranja (zanahoria, calabaza, ahuyama etc.)	☐	☐	☐	☐	☐	☐	☐
Jugos de frutas	☐	☐	☐	☐	☐	☐	☐
Leche baja en grasa (1%, 2%, descremada)	☐	☐	☐	☐	☐	☐	☐
Leche entera (homogenizada)	☐	☐	☐	☐	☐	☐	☐
Queso	☐	☐	☐	☐	☐	☐	☐
Otros productos lácteos (yogur, leche achocolatada, flan, etc.)	☐	☐	☐	☐	☐	☐	☐
Pan de grano integral o cereal integral (avena, Musli, etc.)	☐	☐	☐	☐	☐	☐	☐

CONTROL DE CALIDAD ISCOLE Y DIGITACION DE DATOS:

Iniciales del personal de Control de Calidad: _______________________Fecha: _____ / _____ / 20_____

Iniciales Personal de Digitación: __________________________________Fecha: _____ /_____ / 20_____

ID Participante

Iniciales Encuestador ☐☐☐

Fecha ☐☐ / ☐☐☐ / ☐☐☐☐
Día Mes Año

Cuestionario administrado por: Encuestadora ☐ Auto administrado ☐

	Nunca	Menos de una vez a la semana	Una vez a la semana	2-4 días a la semana	5-6 días a la semana	Una vez al día, todos los días	Todos los días, más de una vez
Sustitutos de la carne (granos, fríjoles,garbanzos, lentejas, tofu, huevos, mantequilla de maní, etc.)	☐	☐	☐	☐	☐	☐	☐
Bebidas energizantes (Red Bull, Rock Star, Guru, Peak, Activade, etc.)	☐	☐	☐	☐	☐	☐	☐
Bebidas deportivas (Gatorade, Powerade, etc.)	☐	☐	☐	☐	☐	☐	☐
Pescado	☐	☐	☐	☐	☐	☐	☐
Helado	☐	☐	☐	☐	☐	☐	☐
Fritos como alas de pollo, dedos de pollo, , empanadas etc.	☐	☐	☐	☐	☐	☐	☐
Comidas rápidas como pizza, hamburguesas, etc.	☐	☐	☐	☐	☐	☐	☐

CONTROL DE CALIDAD ISCOLE Y DIGITACION DE DATOS:

Iniciales del personal de Control de Calidad: _______________________Fecha: _____ / _____ / 20_____

Iniciales Personal de Digitación: _________________________________Fecha: _____ /_____ / 20_____

ID Participante	Iniciales Encuestador ☐☐☐<br>Fecha ☐☐ / ☐☐☐ / ☐☐☐☐<br>Día Mes Año

Cuestionario administrado por: Encuestadora ☐ Auto administrado ☐

34. ¿Usualmente cuántas veces a la semana consumiste los siguientes alimentos **mientras veías televisión**?

Instrucción para la encuestadora:
Con cada opción de pregunta lea todas las opciones de respuesta.
Marque una sola casilla por renglón.

	Nunca	Menos de una vez a la semana	Una vez a la semana	2-4 días a la semana	5-6 días a la semana	Una vez al día, todos los días	Todos los días, más de una vez
Papas fritas de paquete o maní	☐	☐	☐	☐	☐	☐	☐
Fritos como alas de pollo, dedos de pollo, empanadas, papas a la francesa etc.	☐	☐	☐	☐	☐	☐	☐
Galletas, bizcochos, chocolates, caramelos o dulces	☐	☐	☐	☐	☐	☐	☐
Helado	☐	☐	☐	☐	☐	☐	☐
Comidas rápidas como pizza, hamburguesas, etc.	☐	☐	☐	☐	☐	☐	☐
Frutas o verduras	☐	☐	☐	☐	☐	☐	☐

35. ¿Usualmente con qué frecuencia **desayunas** (Lea: desayunar significa comer algo más que un vaso de leche o jugo de fruta)?

Instrucción para encuestadora: Marque una casilla para los días entre semana y una casilla para los fines de semana.

Días entre semana

☐ Nunca desayuno entre semana
☐ Un día
☐ Dos días
☐ Tres días
☐ Cuatro días
☐ Cinco días

Fin de semana

☐ Nunca desayuno los fines de semana
☐ Usualmente desayuno solamente un día del fin de semana (sábado O domingo)
☐ Normalmente desayuno ambos días del fin de semana (sábado Y domingo)

CONTROL DE CALIDAD ISCOLE Y DIGITACION DE DATOS:

Iniciales del personal de Control de Calidad: ______________________Fecha: _____ / _____ / 20_____

Iniciales Personal de Digitación: _________________________________Fecha: _____ /_____ / 20_____

ID Participante	Iniciales Encuestador ☐☐☐<br>Fecha ☐☐ / ☐☐☐ / ☐☐☐☐<br>Día Mes Año

Cuestionario administrado por: Encuestadora ☐ Auto administrado ☐

36. ¿En tu colegio sirven almuerzos?

☐ Sí ☐ No

37. En la última semana que estuviste en el colegio, ¿más o menos **cuántas veces** a la semana almorzaste en el colegio?

☐ 0 días ☐ 1 día ☐ 2 días ☐ 3 días ☐ 4 días ☐ 5 días

38. Durante la semana pasada, ¿cuántas comidas consumiste (Lea**:** desayuno, almuerzo o comida) que hubiesen sido **preparadas fuera de tu casa** en lugares como restaurantes, locales de comida rápida, puestos de comida, tiendas de comestibles o máquinas expendedoras?
(**Lea:** por favor no incluyas comidas ofrecidas como parte del desayuno o almuerzo en tu colegio)

☐☐ Comidas

¿Qué tan bien te describen las siguientes afirmaciones?

Instrucción para encuestadora:
Con cada pregunta lea todas las opciones de respuesta.
Coloque una marca en la casilla que mejor describe lo que ocurre más a menudo.

	Nunca o casi nunca	A veces	Usualmente o siempre
**39.** Como más cuando estoy preocupado	☐	☐	☐
**40.** Como cuando estoy bravo	☐	☐	☐
**41.** Cuando hago algo bien, me premio comiendo algo que me gusta	☐	☐	☐
**42.** Como más cuando me siento triste	☐	☐	☐
**43.** Como más cuando me siento feliz	☐	☐	☐
**44.** Como más cuando estoy aburrido	☐	☐	☐
**45.** Como entre comidas, incluso cuando no tengo hambre	☐	☐	☐

CONTROL DE CALIDAD ISCOLE Y DIGITACION DE DATOS:

Iniciales del personal de Control de Calidad: ______________________Fecha: _____ / _____ / 20_____

Iniciales Personal de Digitación: _________________________________Fecha: _____ /_____ / 20_____

ID Participante	Iniciales Encuestador ☐☐☐ Fecha ☐☐ / ☐☐☐ / ☐☐☐☐ Día Mes Año

Cuestionario administrado por: Encuestadora ☐ Auto administrado ☐

Pensando en la semana pasada…

Instrucción para encuestadora:

Coloque una marca en la casilla que mejor describe cómo se sintió la semana pasada.
Con cada pregunta lea todas las opciones de respuesta.

	**No**, en lo absoluto	Un poco	Moderadamente	Bastante	Extremadamente
**46.** ¿Te sentiste en forma y bien?	☐	☐	☐	☐	☐
**47.** ¿Te sentiste lleno de energía?	☐	☐	☐	☐	☐
**48.** ¿Te sentiste triste?	☐	☐	☐	☐	☐
**49.** ¿Te sentiste solo?	☐	☐	☐	☐	☐
**50.** ¿Tuviste el tiempo suficiente para ti mismo?	☐	☐	☐	☐	☐
**51.** ¿Pudiste hacer las cosas que quieres hacer en tu tiempo libre?	☐	☐	☐	☐	☐
**52.** ¿Te trataron justamente tus padres?	☐	☐	☐	☐	☐
**53.** ¿Te divertiste con tus amigos?	☐	☐	☐	☐	☐
**54.** ¿Te fue bien en el colegio?	☐	☐	☐	☐	☐
**55.** ¿Has podido poner atención?	☐	☐	☐	☐	☐

56. En general, ¿cómo describirías tu estado de salud?

☐Excelente ☐ muy bueno ☐ bueno ☐ regular ☐ malo

CONTROL DE CALIDAD ISCOLE Y DIGITACION DE DATOS:

Iniciales del personal de Control de Calidad: ______________________Fecha: _____ / _____ / 20_____

Iniciales Personal de Digitación: ________________________________Fecha: _____ /_____ / 20_____

ID Participante	Iniciales Encuestador ☐☐☐ Fecha ☐☐ / ☐☐☐ / ☐☐☐☐ Día Mes Año

Cuestionario administrado por: Encuestadora ☐ Auto administrado ☐

Redes de amistad en los colegios.

57. Por favor cuéntame el nombre y apellido de tu mejor amigo(a) del curso o salón, es decir el amigo(a) con el que más tiempo pasas.

Instrucción para encuestadora: La idea es conocer el mejor amigo de cada niño.

____________________________________ ______________

Nombre Apellido ID de ISCOLE

58. Por favor cuéntame el nombre y apellido de tus amigos del curso o salón con los que más tiempo pasas.

Instrucción para encuestadora: La idea es conocer los amigos más cercanos de cada niño y no les damos un número límite de amigos para que la red no sea sesgada.

- ____________________________________ ______________
  Nombre Apellido ID de ISCOLE
- ____________________________________ ______________
  Nombre Apellido ID de ISCOLE
- ____________________________________ ______________
  Nombre Apellido ID de ISCOLE
- ____________________________________ ______________
  Nombre Apellido ID de ISCOLE
- ____________________________________ ______________
  Nombre Apellido ID de ISCOLE
- ____________________________________ ______________
  Nombre Apellido ID de ISCOLE
- ____________________________________ ______________
  Nombre Apellido ID de ISCOLE
- ____________________________________ ______________
  Nombre Apellido ID de ISCOLE
- ____________________________________ ______________
  Nombre Apellido ID de ISCOLE
- ____________________________________ ______________
  Nombre Apellido ID de ISCOLE

Gracias

CONTROL DE CALIDAD ISCOLE Y DIGITACION DE DATOS:

Iniciales del personal de Control de Calidad: _______________________Fecha: _____ / _____ / 20_____

Iniciales Personal de Digitación: _________________________________Fecha: _____ /_____ / 20_____

ID Participante	Iniciales Encuestador ☐☐☐<br>Fecha ☐☐ / ☐☐☐ / ☐☐☐☐<br>Día Mes Año

Cuestionario administrado por: Encuestadora ☐ Auto administrado ☐

APÉNDICE H: Formulario de recopilación de datos antropométricos de ISCOLE

Edad del niño _______ años **Género** ☐Masculino ☐ Femenino

**1. Estatura de pie**	**2.Estatura sentado**	**Estatura total sentado**
1. __ __ __ . __ cm	Altura de mesa/caja:	1. __ __ __ . __ cm
2. __ __ __ . __ cm	1. __ __ __ . __ cm	2. __ __ __ . __ cm
3. __ __ __ . __ cm		3. __ __ __ . __ cm

☐ **Seleccione si el participante no se quitó la moña, hebilla, caimán, balaca u otro elemento para peinarse durante la medición de talla**

**3. Circunferencia del brazo**	**4.Circunferencia de la cintura**
1. __ __ . __ cm	1. __ __ . __ cm
2. __ __ . __ cm	2. __ __ . __ cm
3. __ __ . __ cm	3. __ __ . __ cm

**5. Peso**	**6.Grasa corporal**	**7.Impedancia**
1. __ __ __ . __ kg	1. __ __ . __ %	1. __ __ __ . __ Ω
2. __ __ __ . __ kg	2. __ __ . __ %	2. __ __ __ . __ Ω
3. __ __ __ . __ kg	3. __ __ . __ %	3. __ __ __ . __ Ω

☐ **Seleccione si el participante estaba usando medias/medias veladas durante la toma de peso y porcentaje de grasa corporal.**

CONTROL DE CALIDAD ISCOLE Y DIGITACION DE DATOS:

Iniciales del personal de Control de Calidad: _______________________Fecha: _____ / _____ / 20_____

Iniciales Personal de Digitación: _________________________________Fecha: _____ /_____ / 20_____
