# Supplementary material for: Active streets for children: The case of the Bogotá Ciclovía
Source: PLoS One. 2019 May 15;14(5):e0207791. doi: 10.1371/journal.pone.0207791 (PMC6519789; doi:10.1371/journal.pone.0207791)
Supplement: S7 File — (DOCX) [file pone.0207791.s007.docx]

“Evaluación longitudinal de la diseminación del sobrepeso y actividad física en una red de amistades de una población escolar: la importancia de tecnologías de información y comunicación"

**Instrucción para encuestadora:** Por favor lea cada pregunta completamente. Seleccione la casilla que mejor se ajusta a la respuesta del participante y llénela.

**Leer:** Por favor recuerda que esta encuesta no se trata de un examen, por lo tanto no hay respuestas incorrectas. Es importante que respondas todas las preguntas y que me indiques claramente cuál es tu respuesta. Ninguna persona que te conozca va a ver tu cuestionario o a saber tus respuestas.

Para estas preguntas por favor cuéntame acerca de lo que hiciste la semana pasada.

1. En un día escolar, ¿cuántas horas viste televisión?

⬜ No vi televisión en días escolares. ⬜< 1 hora ⬜ 1 hora ⬜ 2 horas ⬜ 3 horas

⬜ 4 horas ⬜ 5 horas o más

1. En un día escolar, ¿cuántas horas jugaste con juegos de video o de computador, o usaste el computador para cosas que no estén relacionadas con las tareas escolares?

⬜ No jugué con juegos de video o de computador ni usé el computador para cosas que no estuvieran relacionadas con las tareas escolares durante los días de colegio.

⬜< 1 hora ⬜ 1 hora ⬜ 2 horas ⬜ 3 horas ⬜ 4 horas ⬜ 5 horas o más

1. En un día escolar, ¿cuánto tiempo estuviste al aire libre **antes** del colegio?

⬜< 1 hora ⬜ 1 hora ⬜ 2 horas ⬜ 3 horas ⬜ 4 horas ⬜ 5 horas o más

1. En un día escolar, ¿cuánto tiempo estuviste al aire libre **después** del colegio y antes de la hora de dormir?

⬜< 1 hora ⬜ 1 hora ⬜ 2 horas ⬜ 3 horas ⬜ 4 horas ⬜ 5 horas o más

1. En un día de fin de semana, ¿cuántas horas viste televisión?

⬜ No vi televisión en los días de fin de semana. ⬜< 1 hora ⬜ 1 hora ⬜ 2 horas

⬜ 3 horas ⬜ 4 horas ⬜ 5 horas o más

1. En un día de fin de semana, ¿cuántas horas jugaste con juegos de video o de computador, o usaste el computador para cosas que no estuvieran relacionadas con las tareas escolares?

⬜ No jugué con juegos de video o de computador, ni usé el computador para cosas que no estuvieran relacionadas con las tareas escolares durante los días de fin de semana.

⬜< 1 hora ⬜ 1 hora ⬜ 2 horas ⬜ 3 horas ⬜ 4 horas ⬜ 5 horas o más

1. En un día de fin de semana, ¿cuánto tiempo estuviste al aire libre?

⬜< 1 hora ⬜ 1 hora ⬜ 2 horas ⬜ 3 horas ⬜ 4 horas ⬜ 5 horas o más

1. Durante la última semana que fuiste al colegio, ¿cuántos días recibiste clases de educación física (EF)?

⬜ 0 días ⬜ 1 día ⬜ 2 días ⬜ 3 días ⬜ 4 días ⬜ 5 días

1. Durante la última semana que fuiste al colegio, la mayoría de este recorrido lo hiciste:

- caminando
- montando en bicicleta, patines, patineta, monopatín
- en bus, TransMilenio
- automóvil, motocicleta o ciclomotor
- ruta escolar
- por otro medio

1. Durante la última semana que fuiste al colegio, **¿CUÁNTO TIEMPO** te demoraste en el recorrido de tu casa al colegio?

⬜< 5 minutos ⬜ 5 - 15 minutos ⬜ 16 - 30 minutos ⬜ 31 minutos a 1 hora ⬜>1 hora

1. Durante el año pasado (12 meses), ¿realizaste alguna de las siguientes actividades?

**Instrucción para encuestadora:** Marque todas las que apliquen

⬜ Deportes en equipo ⬜ clases de danza o artes marciales ⬜ clases de arte o música

⬜Ninguna de las anteriores

1. Durante la semana pasada (7 días), ¿cuántos días fuiste físicamente activo por lo menos durante 60 minutos diarios?

**Instrucción para encuestadora. Lea**: Piensa en todo el tiempo que estuviste haciendo actividades que incrementaran los latidos de tu corazón y que te hicieran respirar fuerte.

⬜ 0 días ⬜ 1 día ⬜ 2 días ⬜ 3 días ⬜ 4 días ⬜ 5 días ⬜ 6 días ⬜ 7 días

**Instrucción para encuestadora. Lea:**

Por favor dime la opción que más se ajuste a tu respuesta, piensa que:

-1es totalmente en desacuerdo

-2 en desacuerdo

-3 ni de acuerdo ni desacuerdo

-4 de acuerdo

-5 totalmente de acuerdo

**Instrucción para encuestadora:** Con cada opción de pregunta lea todas las opciones de respuesta.

|  | **Totalmente**  **en desacuerdo** |  |  |  | **Totalmente de acuerdo** |
| --- | --- | --- | --- | --- | --- |
|  | **1** | **2** | **3** | **4** | **5** |
| 1. Puedo ser físicamente activo durante mi tiempo libre casi todos los días. |  |  |  |  |  |
|  |  |  |  |  |  |
| 1. Puedo pedirle a mis padres u otro adulto que hagan actividad física conmigo |  |  |  |  |  |
|  |  |  |  |  |  |
| 1. Puedo ser físicamente activo durante mi tiempo libre casi todos los días, incluso en lugar de ver TV o jugar con juegos de video. |  |  |  |  |  |
|  |  |  |  |  |  |
| 1. Puedo ser físicamente activo durante mi tiempo libre casi todos los días, incluso si hace mucho calor o frío afuera |  |  |  |  |  |
|  |  |  |  |  |  |
| 1. Puedo invitar a mi mejor amigo a ser físicamente activo conmigo durante mi tiempo libre casi todos los días |  |  |  |  |  |
|  |  |  |  |  |  |
| 1. Puedo ser físicamente activo durante mi tiempo libre casi todos los días, incluso cuando me tengo que quedar en casa. |  |  |  |  |  |
|  |  |  |  |  |  |
|  | **Totalmente**  **en desacuerdo** |  |  |  | **Totalmente de acuerdo** |
| 1. Poseo la coordinación que necesito para ser físicamente activo en mi tiempo libre casi todos los días |  |  |  |  |  |
|  |  |  |  |  |  |
| 1. Puedo ser físicamente activo durante mi tiempo libre casi todos los días sin importar qué tan ocupado esté mi día |  |  |  |  |  |

**Instrucción para encuestadora. Lea:**

Existen muchas razones por las que la gente hace actividad física. Por favor dime que tanto aplican las siguientes razones en tu caso.

**Instrucción para encuestadora:** Con cada opción de pregunta lea todas las opciones de respuesta.

|  | nunca es cierto en mi caso | algo cierto en mi caso | algunas veces cierto en mi caso | cierto en mi caso | muy cierto en mi caso |
| --- | --- | --- | --- | --- | --- |
| 1. Hago ejercicio porque otras personas me dicen que debería hacerlo. |  |  |  |  |  |
|  |  |  |  |  |  |
| 1. Para mí es importante hacer ejercicio con regularidad |  |  |  |  |  |
|  |  |  |  |  |  |
| 1. No veo porqué debo preocuparme por hacer ejercicio |  |  |  |  |  |
|  |  |  |  |  |  |
| 1. Me siento como un perdedor cuando no hago ejercicio en mucho tiempo |  |  |  |  |  |
|  |  |  |  |  |  |
| 1. Encuentro que el ejercicio es una actividad agradable |  |  |  |  |  |

1. ¿Tienes un televisor en tu habitación?

Sí No

1. ¿Usualmente cuántas veces a la semana comes…?

**Instrucción para encuestadora**:

Con cada opción de pregunta lea todas las opciones de respuesta.

Marque una sola casilla por renglón.

|  | Nunca | Menos de una vez a la semana | Una vez a la semana | | 2-4 días a la semana | 5-6 días a la semana | Una vez al día, todos los días | Todos los días, más de una vez |
| --- | --- | --- | --- | --- | --- | --- | --- | --- |
| Frutas |  |  |  | |  |  |  |  |
| Verduras |  |  |  | |  |  |  |  |
| Dulces (dulces/chocolate) |  |  |  | |  |  |  |  |
| Gaseosa regular o bebidas azucaradas |  |  |  | |  |  |  |  |
| Ponqué, bizcochos o donas |  |  |  | |  |  |  |  |
| Gaseosa de dieta o bebidas azucaradas de dieta |  |  |  | |  |  |  |  |
| Papas fritas de paquete |  |  |  | |  |  |  |  |
| Papas a la francesa |  |  |  | |  |  |  |  |
| Verduras color verde oscuro (brócoli, espinaca, acelga, etc.) |  |  |  | |  |  |  |  |
| Verduras color naranja (zanahoria, calabaza, ahuyama etc.) |  |  |  | |  |  |  |  |
| Jugos de frutas |  |  |  | |  |  |  |  |
| Leche baja en grasa (1%, 2%, descremada) |  |  |  | |  |  |  |  |
| Leche entera (homogenizada) |  |  |  | |  |  |  |  |
| Queso |  |  |  | |  |  |  |  |
| Otros productos lácteos (yogur, leche achocolatada, flan, etc.) |  |  |  | |  |  |  |  |
| Pan de grano integral o cereal integral (avena, Musli, etc.) |  |  |  | |  |  |  |  |
|  | Nunca | Menos de una vez a la semana | Una vez a la semana | | 2-4 días a la semana | 5-6 días a la semana | Una vez al día, todos los días | Todos los días, más de una vez |
| Sustitutos de la carne (granos, fríjoles,garbanzos, lentejas, tofu, huevos, mantequilla de maní, etc.) |  |  |  | |  |  |  |  |
| Bebidas energizantes (Red Bull, Rock Star, Guru, Peak, Activade, etc.) |  |  |  | |  |  |  |  |
| Bebidas deportivas (Gatorade, Powerade, etc.) |  |  |  | |  |  |  |  |
| Pescado |  |  |  | |  |  |  |  |
| Helado |  |  |  | |  |  |  |  |
| Fritos como alas de pollo, dedos de pollo, , empanadas etc. |  |  |  | |  |  |  |  |
| Comidas rápidas como pizza, hamburguesas, etc. |  |  | |  |  |  |  |  |

1. ¿Usualmente con qué frecuencia **desayunas** (Lea: desayunar significa comer algo más que un vaso de leche o jugo de fruta)?

**Instrucción para encuestadora**: Marque una casilla para los días entre semana y una casilla para los fines de semana.

| **Días entre semana**  Nunca desayuno entre semana  Un día  Dos días  Tres días  Cuatro días  Cinco días | **Fin de semana**  Nunca desayuno los fines de semana  Usualmente desayuno solamente un día del fin de semana (sábado O domingo)  Normalmente desayuno ambos días del fin de semana (sábado Y domingo) |
| --- | --- |

1. ¿En tu colegio sirven almuerzos?

Sí No

**Pensando en la semana pasada…**

**Instrucción para encuestadora:**

Coloque una marca en la casilla que mejor describe cómo se sintió la semana pasada.

Con cada pregunta lea todas las opciones de respuesta.

|  | **No**, en lo absoluto | Un poco | Moderadamente | Bastante | Extremadamente |
| --- | --- | --- | --- | --- | --- |
| 1. ¿Te sentiste en forma y bien? |  |  |  |  |  |
|  |  |  |  |  |  |
| 1. ¿Te sentiste lleno de energía? |  |  |  |  |  |
|  |  |  |  |  |  |
| 1. ¿Te sentiste triste? |  |  |  |  |  |
|  |  |  |  |  |  |
| 1. ¿Te sentiste solo? |  |  |  |  |  |
|  |  |  |  |  |  |
| 1. ¿Tuviste el tiempo suficiente para ti mismo? |  |  |  |  |  |
|  |  |  |  |  |  |
| 1. ¿Pudiste hacer las cosas que quieres hacer en tu tiempo libre? |  |  |  |  |  |
|  |  |  |  |  |  |
| 1. ¿Te divertiste con tus amigos? |  |  |  |  |  |
|  |  |  |  |  |  |
| 1. ¿Te fue bien en el colegio? |  |  |  |  |  |
|  |  |  |  |  |  |
| 1. ¿Has podido poner atención? |  |  |  |  |  |

1. En general, ¿cómo describirías tu estado de salud?

Excelente muy bueno bueno regular malo

**Cuestionario sobre actitudes frente a Actividad física y Alimentación Saludable**

|  | **No**, en lo absoluto | Un poco | Moderadamente | Bastante | Extremadamente |
| --- | --- | --- | --- | --- | --- |
| Mis compañeros del curso creen que es bueno hacer ejercicio los fines de semana |  |  |  |  |  |
|  |  |  |  |  |  |
| Mis compañeros del curso creen que es importante comer frutas y verduras |  |  |  |  |  |
|  |  |  |  |  |  |
| Mis compañeros del curso creen que está bien comer paquetes y gaseosas |  |  |  |  |  |
| Mis compañeros del curso piensan que es divertido hacer ejercicio en el recreo |  |  |  |  |  |
|  |  |  |  |  |  |
| La mayoría de mis amigos creen que es bueno hacer ejercicio los fines de semana |  |  |  |  |  |
|  |  |  |  |  |  |
| La mayoría de mis amigos creen que es importante comer frutas y verduras |  |  |  |  |  |
|  |  |  |  |  |  |
| La mayoría de mis amigos creen que está bien comer paquetes y gaseosas |  |  |  |  |  |
|  |  |  |  |  |  |
| La mayoría de mis amigos piensan que es divertido hacer ejercicio en el recreo. |  |  |  |  |  |
|  |  |  |  |  |  |
| Yo creo que es bueno hacer ejercicio los fines de semana |  |  |  |  |  |
|  |  |  |  |  |  |
|  | **No**, en lo absoluto | Un poco | Moderadamente | Bastante | Extremadamente |
| Yo creo que es importante comer frutas y verduras |  |  |  |  |  |
|  |  |  |  |  |  |
| Yo creo que está bien comer paquetes y gaseosas |  |  |  |  |  |
|  |  |  |  |  |  |
| Yo creo que es divertido hacer ejercicio en el recreo |  |  |  |  |  |

**CUESTIONARIO SOBRE CICLOVÍAS**

**Instrucción para encuestadora Lea:**

Donde se menciona al “niño”, por favor responda únicamente acerca del niño que está participando en este estudio. Sea lo más preciso que pueda. Ninguna respuesta es correcta o incorrecta. Toda la información es estrictamente confidencial.

**1.** ¿Con qué frecuencia asistes a la Ciclovía? **(Leer, R.U.)**

| Por lo menos una vez al año | 01 |
| --- | --- |
| 1 día/mes | 02 |
| 2 días/mes | 03 |
| 3 días/mes | 04 |
| 4 días/mes | 05 |
| Siempre | 06 |
| Nunca va a la ciclovia | 07 |

**Instrucción para la encuestadora:** Si contesta nunca pasar a cuestionario módulo M.A.R.A.

1. ¿Qué actividades realizas **usualmente** cuando vas a la Ciclovía? **MR.**

| Montar en bicicleta | 1 |
| --- | --- |
| Patinar | 2 |
| Caminar | 3 |
| Trotar | 4 |
| Montar en scooter | 5 |
| Montar en patineta | 6 |
| Otro, ¿Cuál? | |

1. ¿Con quién vas **usualmente** a la Ciclovía?

| Padres | 1 |
| --- | --- |
| Hermanos | 2 |
| Amigos | 3 |
| Otros familiares | 4 |
| Otro ¿Cual? | |

1. ¿En promedio de horas, cuánto tiempo permaneces en la Ciclovía usualmente?______ MINUTOS
2. ¿En promedio de horas, cuánto tiempo permaneces en la Ciclovía realizando la o las actividades que mencionaste en la pregunta 4? ______ MINUTOS

**CUESTIONARIO MÓDULO M.A.R.A.**

**1.** Con qué frecuencia hiciste actividad física en el recreo durante los últimos siete días (correr, jugar fuerte, saltar, etc.)?

⬜ No hice ⬜ Casi nunca ⬜ Algunas veces ⬜ Casi siempre ⬜ Siempre

**2.** En los últimos 7 días cuál de estas actividades hiciste con mayor frecuencia en el recreo? **(R.U.)**

⬜ Comer

⬜ Estar sentado (hablando, leyendo, haciendo trabajos del colegio)

⬜ Estar parado o caminando

⬜ Correr y jugar un poco

⬜ Correr y jugar alguna parte del tiempo

⬜ Correr y jugar la mayor parte del tiempo

**3.** Cuántas veces participaste en juegos o actividades deportivas en tu tiempo libre durante la última semana (después del colegio, en las tardes o en los fines de semana)

⬜ Ninguna

⬜ 1 vez en la última semana

⬜ 2 o 3 veces en la última semana

⬜ 4 o 5 veces en la última semana

⬜ 6 o 7 veces en la última semana.

**4.** A continuación encontrarás una lista de posibles razones para no hacer actividad física durante el recreo. Léelas cuidadosamente y escoge aquellas que describan de mejor manera las razones por las cuales es difícil hacer actividad física en el recreo en tu colegio (puedes escoger tantas como quieras).

⬜ Porque hace mucho frío afuera

⬜ Porque hace mucho calor afuera

⬜ Porque el patio de recreo es muy pequeño

⬜ Porque es peligroso jugar durante el recreo

⬜ Otra? Cuál? ­­­­­­­­­­­­

⬜ Ninguna

**APÉNDICE H: Formulario de recopilación de datos antropométricos de ISCOLE**

**Edad del niño** _______ años **Género** ⬜Masculino ⬜ Femenino

**1. Estatura de pie**

1. └─┘└─┘└─┘.└─┘cm

2. └─┘└─┘└─┘.└─┘cm

3. └─┘└─┘└─┘.└─┘cm

**2. Circunferencia de la cintura 3.Pliegue del tríceps 4.Pliegue de la pierna**

1. └─┘└─┘.└─┘cm 1. └─┘└─┘.└─┘cm 1. └─┘└─┘.└─┘cm

2. └─┘└─┘.└─┘cm 2. └─┘└─┘.└─┘cm 2. └─┘└─┘.└─┘cm

3. └─┘└─┘.└─┘cm 3. └─┘└─┘.└─┘cm 3. └─┘└─┘.└─┘cm

**3. Peso 4.Grasa corporal 5. Impedancia 6. IMC**

1. └─┘└─┘.└─┘kg 1. └─┘└─┘.└─┘% 1.└─┘└─┘└─┘.└─┘ Ω 1. └─┘└─┘.└─┘kg/m^2^

2. └─┘└─┘.└─┘kg 2. └─┘└─┘.└─┘% 2. └─┘└─┘└─┘.└─┘ Ω 2. └─┘└─┘.└─┘kg/m^2^

3. └─┘└─┘.└─┘kg 3. └─┘└─┘.└─┘% 3. └─┘└─┘└─┘.└─┘ Ω 3. └─┘└─┘.└─┘kg/m^2^
